# DNA methylation-activated full-length EMX1 facilitates metastasis through EMX1-EGFR-ERK axis in hepatocellular carcinoma

**DOI:** 10.1038/s41419-023-06293-y

**Published:** 2023-11-25

**Authors:** Dong-sheng Wen, Li-chang Huang, Xiao-yun Bu, Min-ke He, Zhi-cheng Lai, Ze-feng Du, Ye-xing Huang, Anna Kan, Ming Shi

**Affiliations:** 1 Department of Hepatobiliary Oncology, Sun Yat-sen University Cancer Center, State Key Laboratory of Oncology in South China, Guangdong Provincial Clinical Research Center for Cancer, Guangzhou, 510060 P. R. China; 2 Department of Colorectal Surgery, Hunan Cancer Hospital and the Affiliated Cancer Hospital of Xiangya School of Medicine, Central South University, Changsha, 410013 P. R. China

**Keywords:** Liver cancer, Oncogenes, Growth factor signalling, Cell invasion

## Abstract

Altered DNA methylation is a crucial epigenetic event in hepatocellular carcinoma (HCC) development and progression. Through methylation-transcriptomic analysis, we identified a set of sixty potential DNA methylation-based epidriver genes. In this set of genes, we focused on the hypermethylation of EMX1, which is frequently observed in hepatobiliary tumors. Despite of its frequent occurrence, the function of EMX1 remains largely unknown. By utilizing bisulfite-next-generation sequencing, we have detected EMX1 DNA hypermethylation on the gene body, which is positively correlated with EMX1 mRNA expression. Further analysis revealed that EMX1 mRNA terminal exon splicing in HCC generated two protein isoforms: EMX1 full length (EMX1-FL) and alternative terminal exon splicing isoform (EMX1-X1). Cellular functional assays demonstrated that gain-of-function EMX1-FL, but not EMX1-X1, induced HCC cells migration and invasion while silencing EMX1-FL inhibited HCC cells motility. This result was further validated by in vivo tumor metastasis models. Mechanistically, EMX1-FL bound to EGFR promoter, promoting EGFR transcription and activating EGFR-ERK signaling to trigger tumor metastasis. Therefore, EGFR may be a potential therapeutic target for EMX1-high expression HCC. Our work illuminated the crucial role of gene body hypermethylation-activated EMX1-FL in promoting tumorigenesis and metastasis in HCC. These findings pave the way for targeting the EMX1-EGFR axis in HCC tumorigenicity and metastasis.

## Introduction

Hepatocellular carcinoma (HCC) is considered one of the most fatal cancers and the fastest increasing malignancy [1]. Even though advanced therapies in regional or systemic have been developed [2], the 5-year survival rate for overall HCC remains low (~20%) [1]. To a great extent, the dismal outcomes can be attributed to the high incidence of intra- and extrahepatic metastasis [3]. Finding novel potential molecular targets that could decelerated HCC metastasis is therefore an urgent matter.

The landscape of genome-wide DNA methylation has proven to be a reliable biomarker for HCC epigenetic subtyping and classification [4]. Methylation analysis of tumor tissue DNA or circulating tumor DNA has been shown to be an effective clinical prognostic predictor in primary and recurrence HCC [5–8]. However, the mechanisms of methylation-based epidrivers in HCC remain unclear. While many studies focused on DNA methylation on gene promoters, methylation occurred throughout the genome, including neighboring CGI shores and shelves, intergenic non-coding regions, and gene bodies [9]. Additionally, gene body methylation has been observed to be positively correlated with gene expression, chromatin modifications and RNA alternative splicing, though its function and targeting properties are largely unknown [9–12]. A recent study revealed that ADAMTSL5 overexpression in HCC was positively linked with gene body hypermethylation and conferred tumorigenesis and drug resistance [13]. These findings suggest that more targeted therapies might be discovered by re-evaluating the relationship between gene methylation and expression regulation and by re-evaluating the mechanisms of DNA methyltransferase inhibitors (DNMTi) in HCC treatment [14]. Further investigation into HCC DNA methylation-based epidrivers, particularly in the gene body regions, is therefore a promising field.

Under normal circumstances, the Empty Spiracles Homeobox genes, which include paralog gene EMX1 and EMX2, are restricted in the developmental period of the cerebral cortex [15]. EMX1 is deemed as a marker for pyramidal neurons in the cerebral cortex [16] and widely used in the *Cre-LoxP* system to target neurons [17, 18]. Downregulation of the EMX2 gene has been linked to the development of several solid tumors, such as gastric cancer, lung cancer, endometrial cancer and colorectal cancer, while restoration of EMX2 suppresses cancer progression [19–23]. However, the biological function and detailed molecular mechanism of EMX1 in cancer remain unclear. Despite a few studies focused on EMX1 DNA hypermethylation in hepatobiliary tumors recently [6, 24], its underlying mechanisms have not yet been elucidated.

Here, we have identified EMX1 as a DNA epigenetically upregulated oncogene in HCC. Our analysis revealed an unreported EMX1 3’ splice variant. Specifically, while the EMX1 full length (EMX1-FL) promotes HCC migration and invasion in vitro and metastasis in vivo, this is not observed with the EMX1-X1 (an alternative terminal exon splicing isoform). Additionally, we demonstrated that EMX1-FL activated the transcription of EGFR and upregulated the EGFR-ERK signaling. As a result, this study highlights EMX1 as both the HCC epidriver marker and a potential therapeutic target.

## Results

### EMX1 gene body is hypermethylated in HCC

**Fig. 1:**
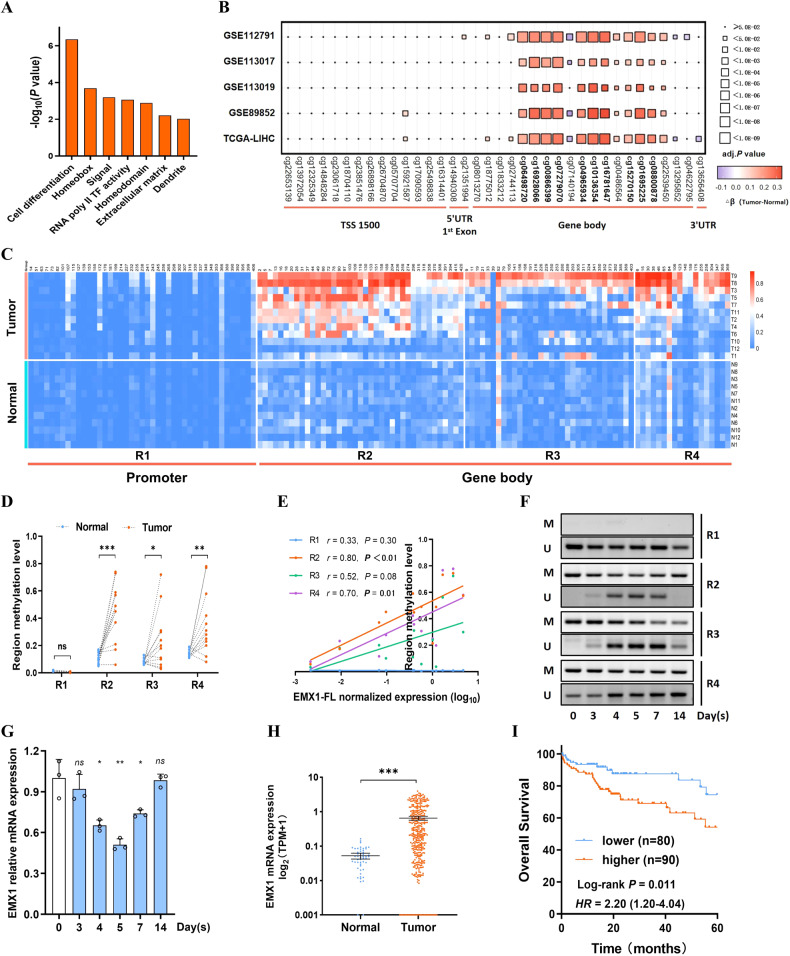
EMX1 is an epidriver and its gene body hypermethylation enhanced EMX1 expression in HCC. **A** The DAVID functional annotation chart of sixty potential DNA epidriver genes. **B** The methylation sites of EMX1 were compared between tumor and ANLT in HCC methylation datasets (GSE112791, GSE113017, GSE113019, GSE89852 and TCGA-LIHC). The top ten most different probes were highlighted (cg06498720, cg16928066, cg00866399, cg07279070, cg04965934, cg10136354, cg16781647, cg15270150, cg01695225 and cg08800878; adj.*P* < 0.0001 and |β_tumor_-β_normal_ |> 0.25). **C** The methylation status of individual CpG sites was quantified by BSP sequencing in HCC tumors and ANLT from R1 to R4 (*n* = 12). R1 is located in the promoter region, R2–R4 are located in the gene body. R1–R4 consist of 44, 44, 42 and 23 CpG sites, respectively. **D** The overall methylation status from R1 to R4 in BSP sequencing (*n* = 12). ns, no significant; *P < 0.05; **P < 0.01; ***P < 0.001. **E** Scatterplot of the correlation between EMX1 regions methylation status and mRNA expression (normalized and log_10_ transformed) in HCC tumor (*n* = 12). **F**, **G** SNU-398 cells were treated with Decitabine (10 μM) for 3 days, and withdrawn for an extra 11 days. **F** DNA methylation status in R1–R4 was quantified using MSP (M, methylated; U, unmethylated). **G** Quantification of relative EMX1 mRNA expression using qRT-PCR. Data shown represent the means (±SD) of three biological replicates. ns, no significant; *P < 0.05; **P < 0.01. **H** The EMX1 total mRNA expression was elevated in HCC tumor tissues derived from the TCGA-LIHC cohort. **I** Kaplan–Meier analysis of overall survival in early-stage HCC (AJCC Stage1, *n* = 170) from TCGA-LIHC cohort based on EMX1 gene expression.

To screen out HCC potential DNA methylation driver genes, we analyzed the TCGA-LIHC DNA methylome and transcriptome. Finally, 60 potential epidriver genes were selected (Supplementary Fig. S1A). These epidrivers mainly enriched in cell differentiation and Homeobox genes (Fig. 1A). Among the genes, six Homeobox family genes (EMX1, OTX1, HOXA10, PITX1, TLX1 and DLX5) consistently expressed higher in HCC tumors (Supplementary Fig. S1B). Four genes (OTX1, HOXA10, TLX1 and DLX5) were reported as oncogenes in multiple tumors [25–28], while PITX1 was extensively reported as tumor suppressor gene [29, 30]. Remarkably, EMX1 was recognized recently as a potential clinically available epi-marker in hepatobiliary tumors [6, 24], but with undefined mechanism and function in HCC.

Next, we assembled four additional methylation validation datasets (GSE112791, GSE113017, GSE113019 and GSE89852) along with TCGA-LIHC to further explore EMX1 aberrant methylation in HCC. We identified the most different methylation probes between tumor and adjacent normal liver tissue (ANLT) (Fig. 1B and Supplementary Fig. S1C). Surprisingly, these probes were located in the region of EMX1 CpG island of intron 1, exon 2 and a predictive alternative exon 3 (namely R2, R3 and R4 respectively), but not the promoter (R1) (the genomic positions of R1–R4 are marked in Supplementary Fig. S1D). We further conducted bisulfite sequencing PCR (BSP) in 12 paired fresh HCC tumors and ANLT on R1~R4 for confirmation (Fig. 1C). The methylation CpG sites that significantly amplified located in R2 (*P* < 0.001), while R3 (*P* < 0.05) and R4 (*P* < 0.01) also showed comparatively higher methylation in tumor. The promoter (R1), however, showed unmethylated status in both tumor and ANLT (Fig. 1D). Methylation-specific PCR (MSP) assay in HCC tumor and ANLT further supported these findings (Supplementary Fig. S1E). We then analyzed the DNA methylation profile of matched samples including primary HCC tumor, portal vein tumor thrombus and ANLT from 20 HCC patients. Probe cg06498720 (locate in R2) showed the highest methylation in portal vein tumor thrombus, compared to both tumor and ANLT (Supplementary Fig. S1F). Taken together, EMX1 gene body hypermethylation is considered as an epidriver that may contribute to HCC tumorigenesis.

### Hypermethylation in gene body upregulated EMX1 expression in HCC

After analyzing BSP sequencing results, we noticed positive correlations between EMX1 gene body methylation and mRNA expression following R2 (*r* = 0.80, *P* < 0.01), R4 (*r* = 0.70, *P* = 0.01) and R3 (*r* = 0.52, *P* = 0.08) in tumor (Fig. 1E). However, correlations were not observed in R1 (*P* = 0.30) and ANLT (Supplementary Fig. S1G). This result was confirmed by TCGA-LIHC and GSE77269 methylation and RNA-seq analyses (https://mexpress.be/index.html and Supplementary Fig. S1H). Then, we conducted DNA demethylation and remethylation experiments by exposure (3 days) and withdrawal (within 2 weeks) of Decitabine (DNMTi) in SNU-398 cells for further verification. MSP analysis indicated that Decitabine increased EMX1 DNA demethylation at R2–R4 at day 3–7, concomitantly, EMX1 mRNA expression decreased during the first week. Conversely, DNA remethylation and EMX1 expression recovered in the withdrawal phase (Fig. 1F, G and Supplementary Fig. S1I). This was further confirmed by methylation bead chip and RNA-seq in SNU-398 and Hep-G2 treated by Guadecitabine (DNMTi) (Supplementary Fig. S1J, K) [14]. These results implied that EMX1 gene body DNA methylation at R2–R4 positively regulates EMX1 expression in HCC.

We then analyzed EMX1 expression level using patient samples. Both TCGA-LIHC cohort (Fig. 1H) and Fudan cohort (Supplementary Fig. S1L) showed that EMX1 total mRNA was significantly upregulated in HCC, while EMX2, an important paralog gene, was barely expressed (data not shown). The TCGA-LIHC iClust subgroups were extracted for further analyses. iClust1 subgroup was previously characterized as a “progenitor-like” subtype with more aggressive clinical features, such as vascular invasion tumors [4]. Interestingly, the expression of EMX1 was significantly higher in iClust1 compared to iClust2&3 (Supplementary Fig. S1M). Besides, higher expression of EMX1 indicated shorter overall survival (*P* = 0.011, TCGA-LIHC cohort, Fig. 1I) and recurrence-free survival (*P* = 0.011, SYSUCC cohort 1, Supplementary Fig. S1N) in early-stage HCC subgroup. In summary, hypermethylation of the EMX1 gene body facilitates EMX1 overexpression and potentially drives HCC tumorigenesis and progression.

### HCC exploits an EMX1 mRNA terminal exon splicing event that regulates its protein nucleus location

**Fig. 2:**
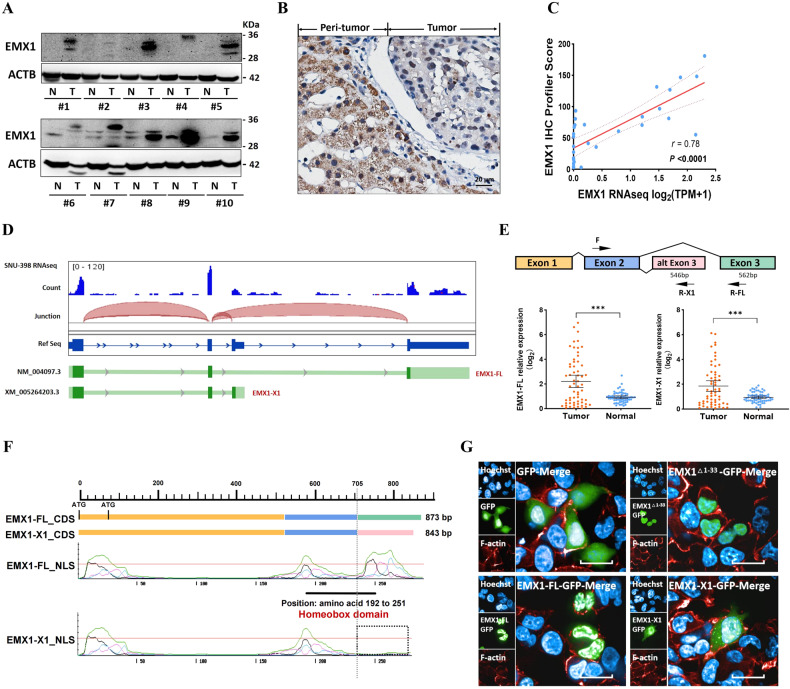
HCC exploits an EMX1 mRNA terminal exon splicing event and regulates its nucleus location. **A** EMX1 multiple isoforms in HCC tumor and ANLT samples detected by western blotting. **B** IHC with anti-EMX1 was performed in HCC samples. Scale bars, 20 μm. **C** Scatterplot of the correlation between EMX1 total mRNA expression (RNA-seq) and total protein (IHC) in the SYSUCC cohort 2. **D** Generation of EMX1 isoforms by terminal exon splicing: EMX1-FL transcript consists of exons 1, 2 and canonical exons 3; EMX1-X1 transcript consists of exons 1, 2 and proximal alternative exons 3; The exons (green) were identified using SNU-398 RNA-seq and spliced alignment analysis. **E** The transcript-specific intron-spanning primers were designed (upper panel) for qRT-PCR and performed on paired HCC tumor and ANLT (SYSUCC cohort 1, *n* = 60). The EMX1-FL expression was showed in lower left panel and the EMX1-X1 expression was showed in lower right panel. The mRNA expression data were shown to represent the means (±95% confidence interval). ***P < 0.001. **F** Graph of two EMX1 transcripts’ coding sequence (CDS) and corresponding nuclear localization signal (NLS) predicted by NLStradamus. The EMX1-FL Homeobox domain is located in amino acid 192–251. **G** Subcellular localization of GFP-tagged vector, EMX1-FL, EMX1-X1 and EMX1^△1-33^ (with a EMX1-FL 1–33 amino acid truncation) shown by immunofluorescence. Phalloidin was used to indicate cytoskeleton. Overlay, merged images of GFP, cytoskeleton and Hochest.

Western blotting was performed to examine the expression of EMX1 targeting the non-Homeodomain sequence (i.e., detecting potential EMX1 different isoforms). The results revealed that besides the prominent band (canonical EMX1-FL, NP_004088, ~31.3 KDa), multiple bands potentially mapped to alternative isoforms were also detected, and EMX1 levels were overall higher in HCC tumor than ANLT (Fig. 2A). Immunohistochemistry (IHC) analyses indicated that EMX1 had different intracellular localization (both cytoplasm and nucleus) in HCC (Fig. 2B). Additionally, we performed IHC and RNA-seq on 30 HCC tissue samples from SYSUCC cohort 2, and verified that the total protein levels of EMX1 were consistent with the total mRNA levels (*r* = 0.78, *P* < 0.0001, Fig. 2C and Supplementary Fig. S2A).

Previous reports have suggested that methylation in the gene body potentially stimulated transcription elongation and splicing outcomes [9, 11]. Upon retrieving expressed sequence tag databases (https://www.ncbi.nlm.nih.gov/IEB/Research/Acembly/) [31], we noticed that region R4 is not only located in EMX1 intron 2 but can also be found on an alternative exon 3 (Supplementary Fig. S1D), indicating that EMX1 mRNA excites terminal exon splicing. Spliced alignment of RNA-seq was therefore performed in SNU-398, and the results showed a proximal alternative third exon that could substitute the third exon, altering the coding region of EMX1 protein at C-terminal (Fig. 2D). Further confirmation was done through 3’RACE sequencing in HCC tumor tissue cDNA (Supplementary Fig. S2B), where it was found that EMX1 mRNA 3’ end had a consensual 562-bp-long terminal exon and a shorter alternative terminal exon (546 bp). Thus, two distinct EMX1 proteins can be generated: a canonical EMX1 full-length (EMX1-FL) isoform and an EMX1 isoform with alternative C-terminal end (EMX1-X1). Then the transcript-specific intron-spanning primers were designed for qRT-PCR (Fig. 2E upper panel and Supplementary Fig. S2C, D). The results from sixty paired samples showed that both of these two EMX1 transcripts were highly expressed in HCC tumor tissue compared to ANLT (Fig. 2E lower panel and Supplementary Fig. S2F), and displayed a high similarity of expression patterns (*r* = 0.95, *P* < 0.0001; Supplementary Fig. S2E).

After aligning the sequence, we found that EMX1-FL terminator exon coding part of the Homeodomain (between amino acid 192 to 251) along with the nuclear localization signals (NLS) [32] (Fig. 2F). Since annotation results suggesting that EMX1 might shuttle between nucleus and cytoplasm, we investigated whether the alternative terminator splicing (by truncating the terminal NLS) or potential alternative translation initial sites (ATG^34^, by truncating the initial NLS) regulated its pivotal nuclear location. We constructed GFP-tagged control vector, EMX1-FL, EMX1-X1 and truncated EMX1^Δ1-33^, and transfected them into SNU-398. Immunofluorescence assays confirmed the exogenous expression and subcellular localization. EMX1-FL and EMX1^Δ1-33^ (with entire Homeodomain) were predominantly localized in the nucleus, while EMX1-X1 particularly accumulated in the cytoplasm or vesicles (Fig. 2G).

### EMX1-FL but not its alternative splicing isoform enhances HCC migration and metastatic ability in vitro and in vivo

**Fig. 3:**
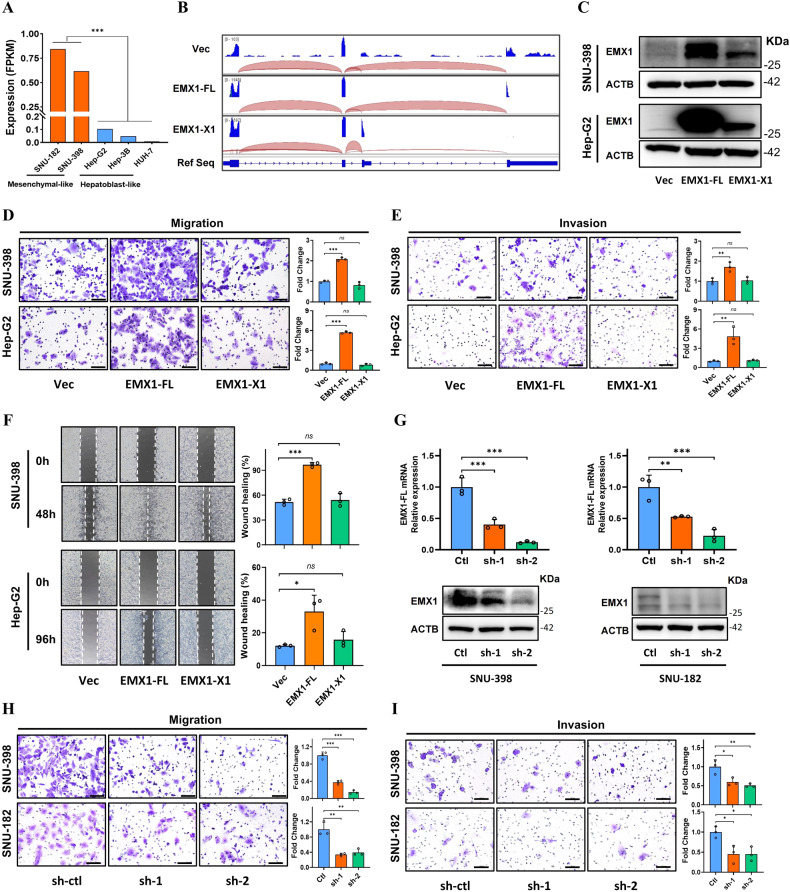
EMX1-FL but not its alternative splicing isoform enhances HCC migration and invasion ability in vitro. **A** HCC cell lines total EMX1 expression by RNA-seq from Liver Cancer Cell Lines Database. **B**, **C** Forced expression of transcript-specific EMX1 in SNU-398 and Hep-G2 and validated by RNA-seq (**B**) and western blotting (**C**). **D**–**F** Representative images and quantification of in vitro migration (**D**), invasion (**E**) and wound healing (**F**) assays in SNU-398 (upper panel) and Hep-G2 (lower panel) cells stably transfected with transcript-specific EMX1 or vector control. Scale bar, 100 μm. **G** Silencing of EMX1 in SNU-398 (left panel) and SNU-182 (right panel) using shRNA lentivirus and validated by qRT-PCR (upper panel) and western blotting (lower panel). **H**, **I** Representative images and quantification of in vitro migration (**H**) and invasion (**I**) assays in SNU-398 (upper panel) and SNU-182 (lower panel) cells stably transfected with EMX1 shRNA or vector control. Scale bar, 100 μm. Data shown represent the means (±SD) of three biological replicates. ns, no significant; *P < 0.05; **P < 0.01; ***P < 0.001.

We started out by investigating the total EMX1 mRNA expression in SNU-182, SNU-398, Hep-G2, Hep-3B and Huh-7 HCC cell lines [33]. Interestingly, compared to the hepatoblast-like cell lines (Hep-G2, Hep-3B and Huh-7), the mesenchymal-like cell lines (SNU-182 and SNU-398) exhibited relatively higher expression of EMX1 (Fig. 3A) [34]. We then established stable EMX1 transcript-specific overexpression in two mesenchymal-like cell lines (SNU-398 and SNU-182) and two hepatoblast-like cell lines (Hep-G2 and Huh-7) as well as knockdown in SNU-182 and SNU-398 to assess the functions of EMX1 in HCC. We confirmed the ectopic EMX1 isoforms expression by qRT-PCR (Supplementary Fig. S3A), RNA-seq (Fig. 3B) and western blotting (Fig. 3C). The ectopic EMX1 isoforms in SNU-398 and Hep-G2 showed similar growth patterns to the vector control in vitro (Supplementary Fig. S3B) and in vivo (the subcutaneous xenograft model showed EMX1-FL seemed to grow faster but had no statistical significance; Supplementary Fig. S3C–E), indicating that neither EMX1-FL nor EMX1-X1 contribute to proliferation of HCC cells. We next investigated the role of EMX1 in cell migration and invasion. The results showed that overexpression of EMX1-FL, but not the alternative terminal exon splicing isoform EMX1-X1, significantly promoted SNU-398, SNU-182, Hep-G2 and Huh-7 cells migration, invasion, and wound healing in vitro (Fig. 3D–F and Supplementary Fig. S3F, G). Conversely, EMX1-FL knockdown in SNU-398 and SNU-182 cell lines (Fig. 3G) showed significant weaker migration and invasion (Fig. 3H, I).

**Fig. 4:**
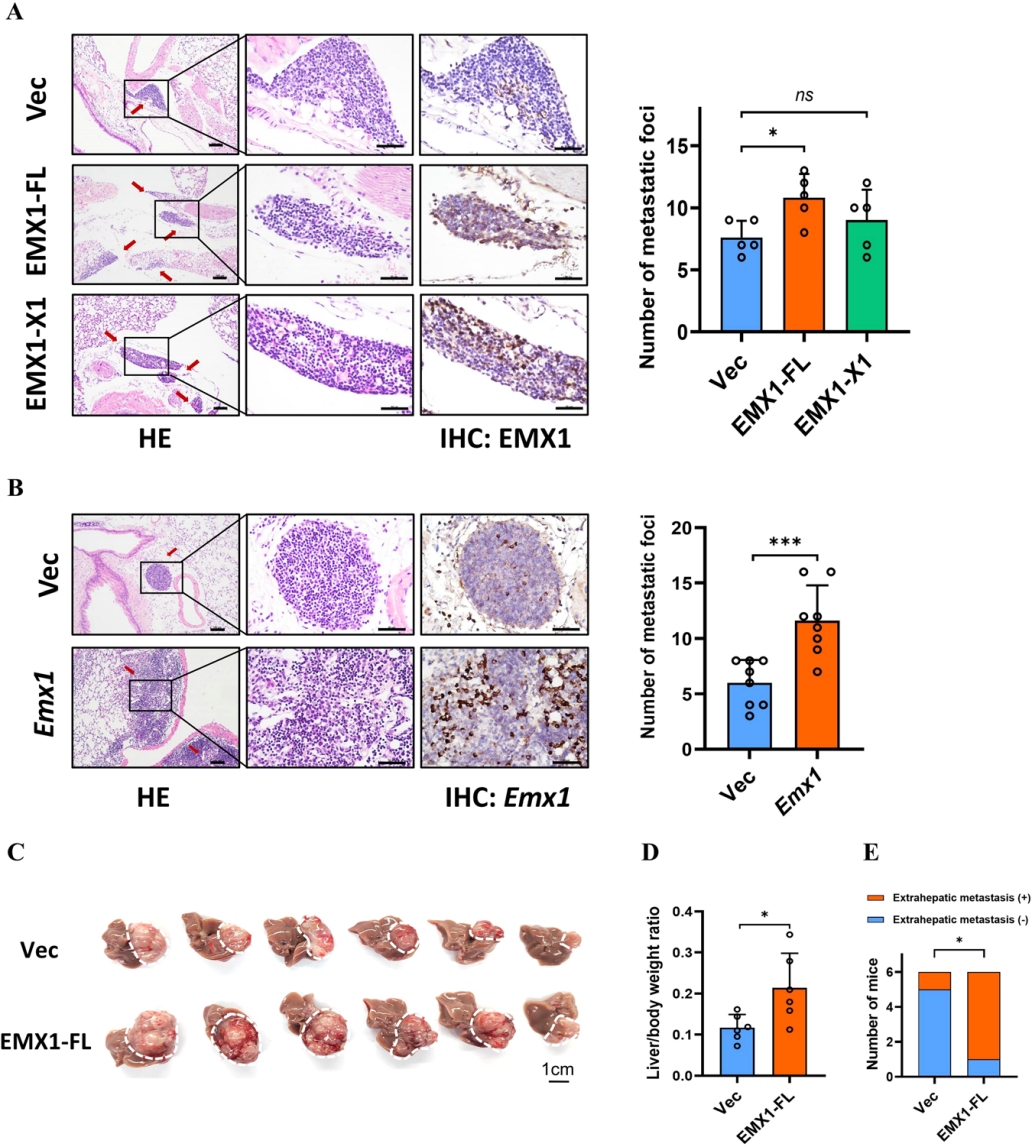
EMX1-FL enhances HCC metastasis in vivo. **A**, **B** The tail-vein injection lung metastasis model: nude mice model (**A**) and the C57BL/6 mice model (**B**). In the left panel, representative images of HE staining (scale bar, 100 μm) and IHC anti-EMX1 staining (scale bar, 50 μm) are shown after the tail-vein injection of **A** SNU-398 vector control, OE-EMX1-FL or OE-EMX1-X1 cells (*n* = 5 per group), **B** Hepa1-6 vector control or OE-*Emx1* cells (*n* = 8 per group). The arrows indicate lung metastasis foci. The right panels show quantification of the number of metastasis foci. **C**–**E** The orthotopic xenograft HCC mouse model. **C** Gross images with the white dotted box indicates intrahepatic tumor nodules. **D** Quantification of the liver/body weight ratio. **E** Quantification of mice with extrahepatic metastases (*n* = 6 per group). Error bars represent the mean ± SD. ns, no significant; *P < 0.05; ***P < 0.001.

To further clarify the functions of EMX1 in HCC in vivo, we injected EMX1-FL and EMX1-X1 overexpressing SNU-398 cells into the nude mice via tail-vein. Histological examination of lung tissues revealed a significant increase in the number of lung metastasis foci in the OE-EMX1-FL group, while no statistical significance was observed in OE-EMX1-X1 group (Fig. 4A). For *Mus musculus*, there is only one validated transcript of *Emx1* (NM_010131.2). Thus, we constructed an *Emx1* overexpressing Hepa1-6 cells model (Supplementary Fig. S4A). The tail-vein lung metastasis model in C57BL/6 also showed that *Emx1* overexpression increased the metastatic foci (Fig. 4B). For orthotopic xenograft implantation HCC model, OE-EMX1-FL promoted vigorously SNU-398 intrahepatic proliferation and extrahepatic metastasis (Fig. 4C–E and Supplementary Fig. S4B, C). To sum up, our findings suggest that EMX1-FL, but not EMX1-X1, promotes HCC migration, invasion and metastasis in vitro and in vivo.

### EMX1-FL promotes EGFR-ERK signaling in HCC

**Fig. 5:**
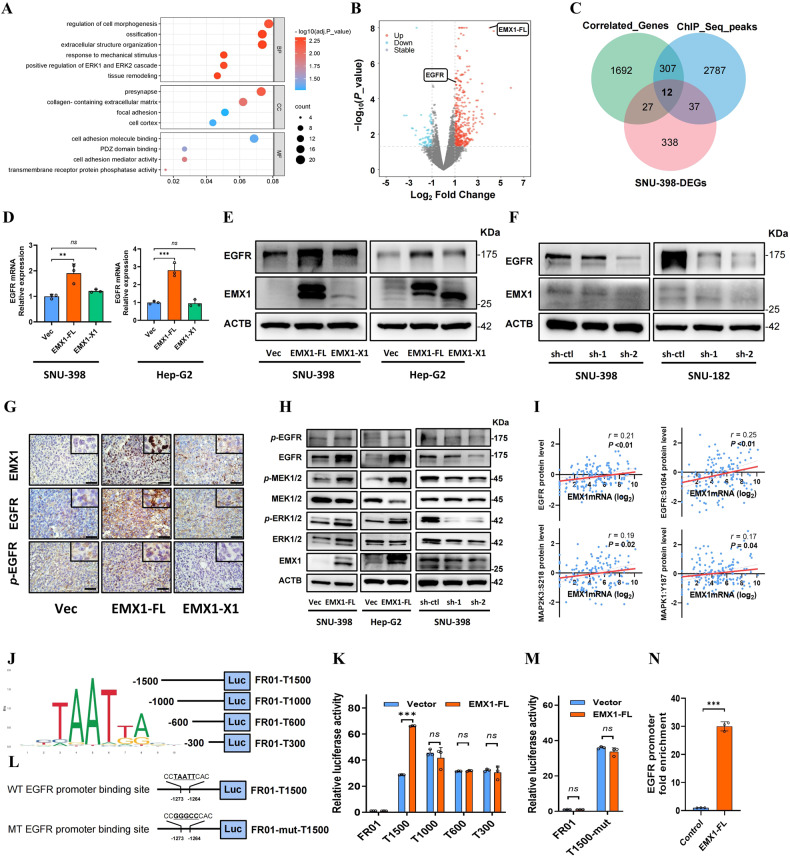
EMX1-FL promotes EGFR-ERK signaling in HCC. **A**, **B** The DEGs of SNU-398 EMX1-FL overexpression were enriched by Gene Ontology analysis (**A**) and shown as a volcano plot (**B**). **C** The joint analyses of SNU-398 DEGs, express-correlated genes in TCGA-LIHC and EMX1 Chip-seq peaks from a public databases (http://cistrome.org/db/). **D**, **E** Ectopic expression of EMX1-FL, but not the EMX1-X1, increased the total EGFR mRNA (**D**) and protein (**E**) levels in SNU-398 and Hep-G2. **F** EMX1 Silencing decreased the total EGFR protein level in SNU-398 and SNU-182. **G** Ectopic expression of EMX1-FL, but not the EMX1-X1, increased the total EGFR and *p*-EGFR (Tyr1068) protein in the subcutaneous xenograft model detected by IHC. **H** EMX1-FL overexpression (SNU-398 and Hep-G2) activated EGFR-ERK signaling, while silencing (SNU-398) repressed EGFR-ERK signaling. **I** Scatterplots of the correlation between EMX1 mRNA expression and total protein of EGFR, *p*-EGFR (S1064), *p*-MAP2K3(S218) and *p*-MAPK1(Y187) in the Fudan cohort. **J** According to the Homeodomain rich frequency matrix (left panel), four truncations of EGFR promoter were constructed into the pEZX-FR01 luciferase reporter plasmids (right panel). **K** Relative promoter activity of truncations in SNU-398 luciferase reporter assays. **L** Wild type and mutant binding site on the EGFR promoter. **M** Relative activity of mutant promoter in SNU-398 luciferase reporter assays. **N** The CUT&Tag-qPCR assay measured the occupancy of EMX1-FL on the promoter of EGFR in SNU-398. Data shown represent the means (±SD) of three biological replicates. ns, no significant; **P < 0.01; ***P < 0.001.

EMX1-FL embeds an entire sequence-specific DNA binding Homeodomain, which is predicted to regulate gene transcription. However, the precise downstream pathway and target genes have not been identified. We performed Gene Ontology analysis upon EMX1-FL overexpressing SNU-398 and found that differentially expressed genes (DEGs) were mainly enriched in cell adhesion and migration-associated pathways (Fig. 5A). The metastasis of cancers often relies on cell-surface receptors specific to various growth factors [35]. Interestingly, we also noticed that transmembrane receptor protein phosphatase and associated ERK1/2 cascade were significantly upregulated in EMX1-FL overexpressing cells. It was also worth noting that analysis on EMX1-X1 overexpressing SNU-398 showed enrichment in pathways regulating vasculature development, angiogenesis, mesenchymal cell differentiation, stem cell development and so on (Supplementary Fig. S5A, B). Through integrating the EMX1-FL DEGs, express-correlated genes and EMX1 Chip-seq peaks in public dataset (Fig. 5B, C), we identified EGFR, which also was the hub gene in our DEG network (Supplementary Fig. S5C). We next assumed that whether EGFR is the key downstream of EMX1-FL.

We detected EGFR mRNA and protein levels and found significantly increased under EMX1-FL ectopic expression, but not in the EMX1-X1 (Fig. 5D, E). Conversely, EMX1-FL knockdown showed the opposite results in SNU-398 and SNU-182 (Fig. 5F). We further confirmed the protein levels in vivo, showing that EMX1-FL promotes EGFR and *p*-EGFR (Tyr1068) expression in the subcutaneous xenograft model (Fig. 5G). The expression levels of *p*-MEK1/2, *p*-ERK1/2 were also increased in EMX1-FL overexpressing SNU-398, Hep-G2 and Huh-7, while the opposite was observed in EMX1-FL knockdown SNU-398 (Fig. 5H and Supplementary Fig. S5D). Correspondingly, we observed a significant positive expression correlation between EMX1 mRNA and EGFR-ERK signaling in HCC in Fudan cohort (Fig. 5I), TCGA cohort and our SYSUCC cohort 1, but not in ANLT samples (Supplementary Fig. S5E). These results suggested that EMX1-FL plays a positive regulative role in EGFR-ERK signaling in HCC.

To identify the potential direct transcriptional regulation of EMX1 on the EGFR promoter, we generated a series of truncated EGFR promoters according to predicted binding sites (Fig. 5J). Luciferase reporter assays suggested that the truncated promoters exhibited lower activity compared to the full-length promoter length (1.5 Kb) (Fig. 5K), indicating the region between –1500 and –1000 bp upstream of the transcription start site (TSS) was necessary for EMX1-FL binding to EGFR. We then generated an EGFR promoter with a mutated binding site at –1273 to –1264 bp upstream of the TSS (Fig. 5L). The mutation abolished EMX1-FL mediated transcriptional activity (Fig. 5M), indicating that EMX1-FL directly bonded to this site to regulate the transcription of EGFR. Further CUT&Tag sequencing (Supplementary Fig. S5F) and CUT&Tag-qPCR (Fig. 5N) also showed that EMX1-FL was remarkably enriched on the EGFR promoter. In conclusion, we demonstrated that EMX1-FL directly activates EGFR transcription and upregulates EGFR-ERK signaling.

### EMX1-FL promotes metastasis via EGFR signaling, and EGFRi reverses the effects of overexpressing EMX1-FL

**Fig. 6:**
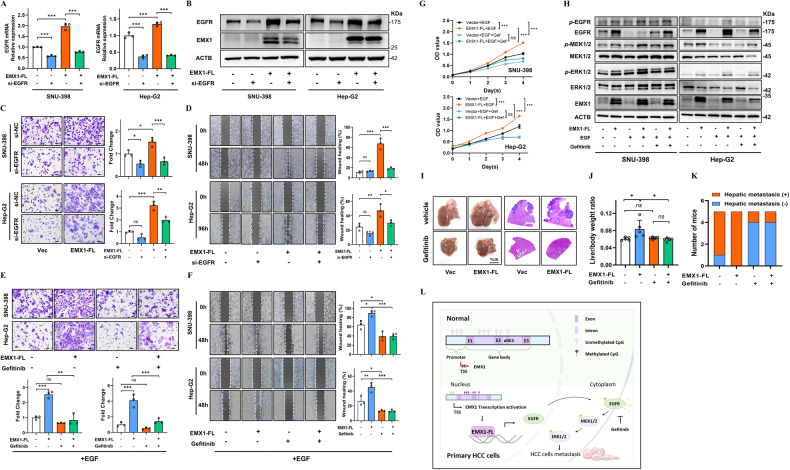
EMX1-FL promotes HCC metastasis via EGFR signaling. **A**, **B** siRNA showed partial silencing of EGFR mRNA (**A**) and protein (**B**) in SNU-398 (left panel) and Hep-G2 (right panel). **C**, **D** Representative images and quantification of migration (scale bar, 50 μm) (**C**) and wound healing (**D**) assays in SNU-398 (upper panel) and Hep-G2 (lower panel), with silenced EGFR or not in vector control and OE-EMX1-FL. **E**–**G** Representative images and quantification of migration (scale bar, 50 μm) (**E**), wound healing (**F**) and proliferation (**G**) assays in SNU-398 (upper panel) and Hep-G2 (lower panel), treated with Gefitinib (10 μM) or not in vector control and OE-EMX1-FL (all cells were pre-treated with 20 ng/ml EGF). **H** The western blotting detected EGFR-ERK signaling in SNU-398 and Hep-G2 cells treated with or without Gefitinib (10 μM) in vector control and OE-EMX1-FL (with or without 20 ng/ml EGF). **I**–**K** The intrasplenic injection HCC orthotopic model. **I** Gross images (left panel) and representative images of HE staining (right panel) of mice livers. **J** Quantification of liver/body weight ratio (*n* = 5 per groups). **K** Quantification of mice with intrahepatic metastases. **L** Graphical abstract of EMX1-EGFR signaling in HCC cells. Data shown represent the means (±SD) of biological replicates. ns, no significant; *P < 0.05; **P < 0.01; ***P < 0.001.

To determine whether the regulation of migration by EMX1-FL depended on EGFR signaling, we silenced EGFR in SNU-398 and Hep-G2 by using siRNA. Partial silencing of EGFR (Fig. 6A, B) successfully reversed the cell migration and wound healing capacity upon EMX1-FL overexpression (Fig. 6C, D). The transwell, wound healing and CCK8 assays verified that the addition of exogenous EGF significantly enhanced cellular migration and growth in EMX1-FL overexpressing cells, as compared to vector cells. What’s more, targeting EGFR using Gefitinib (a first-generation EGFR inhibitor) offset the growth-promoting effect, as well as the migration-promoting effect caused by the addition of exogenous EGF in EMX1-FL overexpressing cells (Fig. 6E–G). The immunoblotting revealed that Gefitinib could block the EMX1-FL promoting EGFR-ERK signaling (Fig. 6H). In the intrasplenic injection orthotopic HCC model, we observed high intrahepatic metastasis incidents in both the EMX1-FL overexpressing group (5 of 5) and the control group (4 of 5), while the EMX1-FL overexpressing group showed a higher liver/body weight ratio. These were significantly attenuated after Gefitinib treatment (Fig. 6I–K). In conclusion, our results suggested that EMX1-FL promotes HCC cells migration by activating EGFR transcription and EGFR-ERK signaling, while blocking the EGFR signals can reversed these effects and reduced HCC metastasis (Fig. 6L).

## Discussion

HCC is a highly lethal malignancy with a high potential for metastasis and frequent recurrence [1–3]. Alterations in DNA methylation landscape are key epigenetic events in HCC [4]. In this study, we identified sixty potential epidriver genes. We were especially intrigued by EMX1, a gene previously linked to neurodevelopment. We discovered that hypermethylation of the gene body of EMX1 was associated with HCC tumorigenesis and positively correlated to its expression. However, the promoter region remained largely unmethylated. Recently, Su et al. conducted a pan-cancer analysis and found a correlation between gene body hypermethylation of DNA methylation canyons and overexpression in approximately 43% of Homeobox genes, interestingly, many of which are oncogenes [36]. These findings aligned with our observation in EMX1 and were confirmed by exposing and withdrawing DNMTi in HCC cells. Our results indicated that some of the CpGs located in EMX1 gene bodies may represent functional elements under tumorigenesis circumstances, as they are associated with histone methylation modifications (such as H3K4 and H3K27), and histone (de)methylase enrichment (such as EZH2 and JARID) in HCC tissues and cell lines (Supplementary Fig. S6). However, further studies are required to uncover the molecular details of this regulation.

Transcription factors regulate gene programs, thereby controlling diverse cellular processes [37]. Alternative splicing events can generate cancer-associated transcription factors isoforms with altered activity [38]. We noticed that EMX1 mRNA excites terminal exon splicing, where the alternative third exon substitutes the third exon and the coding region of protein at the C-terminal, which disrupting the Homeodomain NLS. To our knowledge, this alternative splicing event has not been documented in the literature but can be retrieved from some expressed sequence tag databases [31]. The characteristics are often drastically different between splice isoforms of the same transcription factor, and these differences cannot be predicted based on nominal domain annotations or consensus sequence [37]. The advance of results indicated that EMX1-FL, but not its alternative splicing isoform EMX1-X1, is located in the nucleus and enhances HCC migration and metastatic ability in vitro and in vivo. Additionally, we found that EMX1-X1 may be involved in vasculature development and cell differentiation processes in DEGs enrichment analyses. This may be partly contributed to the different terminator in the Homeodomain. Additionally, the link between terminal exon splicing and oncogene altered activity can also be observed through a difference of 3’UTR regions by posttranscriptional regulatory mechanisms [39]. Nonetheless, further studies are required to unravel the EMX1 splicing regulators and the distinct isoforms in cancers, linking the potential relationship between gene body methylation, RNA splicing, and the roles played by EMX1 isoforms.

The human EMX1 mRNA is encoded by the 18.7-kbs EMX1 gene located at 2p13.2. This is not a susceptible locus that frequently changes in copy number or mutates in HCC [4, 40]. Previously, EMX1 was considered to be a tissue-specific gene in the developing brain [15, 16]. The potential of EMX1 in tumorigenesis was not appreciated until Jimenez-Garcia et al. discussed EMX1 and its analog (EMX2) suppressing osteosarcoma progression [41, 42]. They highlighted that EMX1 and EMX2 act as tumor suppressors by restraining the effector of the canonical WNT pathway, though the precise molecular mechanism remained unknown [42]. However, our study suggested that EMX1 could be an oncogene activated by DNA methylation in the development of HCC. Through a series of metastatic assays following EMX1 perturbation, we unexpectedly discovered that EMX1-FL promoted cell migration and invasion in vitro and metastasis in vivo in an isoform-dependent manner. And there was not significant change in WNT pathway under EMX1 perturbation in our system (data not shown). These suggest that EMX1 potentially exhibits both oncogenic and tumor-suppressive activities in a context-dependent manner.

Recent studies have pointed out that EGFR signaling contributed to the essential regulation of malignant biological behaviors of HCC cells, mainly in metastasis, recurrence, and drug resistance [43, 44]. In the present study, we identified EGFR as the crucial target of Homeodomain-dependent EMX1 signaling. This may be supported by a report that *Emx1* null mice neural stem cells reduced migratory capacity in response to serum or vascular endothelial growth factor during brain development [45]. In our in vitro experiments, we found that EMX1-FL overexpression promoted cell migration and invasion, while EMX1-X1 did not. These can be due to the different regulation of EGFR. Interestingly, both two EMX1 transcripts overexpression individually did not demonstrate a significant proliferation effect in vitro. However, in vivo models (the cell line derived orthotopic xenograft mouse model and the intrasplenic injection orthotopic model) suggested that EMX1-FL promoted cell metastasis and proliferation. These discrepancies may be due to differences in the in vivo cellular conditions. Indeed, the cell lines also showed a proliferative response to exogenous EGF. We then demonstrated that EMX1-FL’s promotion of metastatic potential depend on the transcription activation of EGFR, and subsequently upregulating EGFR-ERK signaling. Blocking the EGFR signals by EGFRi (Gefitinib, etc.) can reversed these effects and reduced HCC metastasis. These findings pave the way for targeting the EMX1-EGFR axis in HCC tumorigenicity and metastasis.

There are several limitations in this study. First of all, the EMX1 gene body methylation and expression in this study mainly based on bioinformatic analyses, and whether the promotion of EMX1 was caused by gene body hypermethylation remained unclear as Decitabine is a global hypomethylation agent. The special methylation editing techniques (e.g., dCas9 mediated DNA methylation editing) may be suitable for further investigation. Secondly, we mainly focused on EMX1-FL function in promoting EMX1-EGFR-ERK signaling. However, more results need to be elucidated as to whether the alternative transcript EMX1-X1 also plays a role in HCC tumorigenesis and progression, but specific antibodies against EMX1 isoforms are currently lacking. Thirdly, the utilization of EGFRi to demonstrate the pro-metastasis role of EMX1-FL in HCC dependent on EGFR signaling is insufficient. In our ongoing research, we are currently employing gene editing techniques to specifically mutate the target sequence on the EGFR promoter that binds to EMX1-FL. Finally, targeting EMX1 inhibitor also merits further exploration.

In conclusion, our study revealed the EMX1-EGFR-ERK axis and, for the first time, shed light on the mechanism of EMX1-FL in stimulating HCC metastatic properties, and EMX1-EGFR therefore is a potential target for reducing HCC metastasis.

## Materials and methods

### Patients and public datasets

The study was approved by the Committee for the Ethical Review of Research of Sun Yat-sen University Cancer Center (SYSUCC). Written informed consent was obtained following institutional guidelines and the declaration of Helsinki guidelines. The Cancer Genome Atlas of Liver Hepatocellular Carcinoma (TCGA-LIHC) methylation profile (*n* = 368) and RNA-seq profile (*n* = 371) were obtained from http://www.cbioportal.org. Another five HCC methylation gene sets (GSE112791, GSE113017, GSE113019, GSE89852, GSE77269) were collected from Gene Expression Omnibus (https://www.ncbi.nlm.nih.gov/gds) for verification. The Fudan RNA-seq cohort (*n* = 159) was obtained from Zhongshan Hospital within Fudan University (Shanghai, China) [40]. Additionally, sixty paired fresh-frozen tumors and adjacent normal liver tissue (ANLT) specimens were obtained from pathological diagnosis HCC patients who undergone surgery in SYSUCC for experimental validation (SYSUCC cohort 1). SYSUCC cohort 2 (*n* = 30, RNA-seq) was obtained from HCC tumor biopsies. Details of the patients’ clinical characteristics and high-throughput data analyses are provided in the Supplementary Information.

### Bisulfite-next-generation sequencing PCR (BSP) and methylation-specific PCR (MSP)

Genomic DNA was extracted from 12 paired fresh HCC tumors and ANLT (from SYSUCC cohort 1) using a Ezup Column Animal Genomic DNA Purification Kit (Sangon Biotech, Shanghai, China). The purified DNA was bisulfite-converted using the EZ DNA Methylation-Gold^TM^ Kit (Zymoresearch, USA) according to the manufacturer’s protocol. For BSP, the bisulfite-converted genomic DNA was amplified using specific BSP primers, and PCR products were pooled and subjected to next-generation deep sequencing to determine the methylation status of CpG sites. Sangon Biotech (Shanghai, China) performed library preparation and sequencing. BSP covered four regions of the EMX1 gene, each with different numbers of CpG sites: R1–R4 contained 44, 44, 42 and 23 CpG sites, respectively. MSP of bisulfite-converted DNA was carried out with specific methylated or unmethylated primer. Amplified PCR products were separated by 3% agarose gel electrophoresis and visualized with GelRed. Specific CpG dinucleotides analyzed within each region and the primers used in BSP and MSP were shown in Supplementary Information.

### Mouse experiments

All mouse husbandry and procedures were in accordance with protocols approved by the Institutional Care and Animal Use Committee of SYSUCC. No blinding was used during the experiment procedures.

For the subcutaneous xenograft model, SNU-398 vector control, OE-EMX1-FL or OE-EMX1-X1 (1 × 10^6^ cells per mouse) were injected subcutaneously into the right posterior flanks of 6-week-old male nude mice (*n* = 5 per group). The tumor volume was evaluated based on calliper measurements and calculated using the modified ellipsoidal formula: tumor volume = 1/2 length × width^2^.

For the tail-vein injection lung metastasis model, SNU-398 vector control, OE-EMX1-FL or OE-EMX1-X1 (2 × 10^6^ cells per mouse) were injected into 6-week-old male nude mice via the tail-vein (*n* = 5 per group). The mice were sacrificed after 6 weeks. Hepa1-6 vector control or OE-*Emx1* (2 × 10^6^ cells per mouse) were injected into 6-week-old male C57BL/6 mice via the tail-vein (*n* = 8 per group). Mice were sacrificed after 8 weeks. The murine lungs were perfused with 10 ml of PBS through the right ventricle until they were cleared of blood. The tissues were then rinsed with pre-chilled PBS and fixed in 4% paraformaldehyde overnight, and then embedded in paraffin. H&E staining was then performed on consecutive sections of the whole lung. The metastatic foci were recorded microscopically as the maximum number in one section to assess the development of pulmonary metastasis.

For the cell line derived orthotopic xenograft model, subcutaneous tumors were first established by inoculating SNU-398 vector control or OE-EMX1-FL (1 × 10^6^ cells per mouse) into the right posterior flanks of nude mice. When they grew to 10 mm in diameter, tumors were harvested, and non-necrotic tissues were cut into 1 mm^3^ pieces and then implanted into the liver left lobe of tumor-free male nude mice (*n* = 6 per group). The mice were euthanized after 4 weeks. Liver and body weight were recorded, and the presence of extrahepatic metastasis foci was carefully documented.

For the intrasplenic injection HCC orthotopic model, SNU-398 vector control or OE-EMX1-FL (2 × 10^6^ cells per mouse) were injected into 6-week-old male nude mice (*n* = 10 per group) via intrasplenic injection. After 3 weeks, mice were randomly assigned to 5 days per week treatment with vehicle or Gefitinib (80 mg/kg, oral gavage) (*n* = 5 per group). All mice were euthanized after 2 weeks of drug treatment and 1 week of observation. Body weight was monitored throughout the treatment. Liver and body weight were recorded at the end of experiments, and the livers were then processed for histological examination.

### Drug treatment

Gefitinib (S1025), Decitabine (S1200) were purchased from Selleck Chemicals. For DNA methyltransferase inhibitor assay, adherent SNU-398 cells were treated with 10 μM Decitabine at the onset day (D0). After 3 days (D3), the media were replaced with fresh drug-free media and refreshed every other day for an extra 11 days (until D14). Control cells were cultured under similar experimental conditions in the absence of drugs. For blockade of EGFR activation, cells were treated with Gefitinib (10 μM) for 48 h before sample collection.

### Statistics

All statistical analyses in this study were performed using the GraphPad Prism 8.0 and R 4.0. Two-tailed Student’s *t*-test or one-way ANOVA was applied for calculating statistical significance. The Log-rank test was used for survival analyses. Gene expression correlation was calculated through Pearson correlation analysis. The *P* values < 0.05 were considered statistically significant.

More details for other methods, including cell lines information, 3’ rapid amplification of cDNA ends (3’RACE), plasmids construct, lentivirus infection, transiently silencing or overexpression, RNA extraction and quantitative real-time PCR (qRT-PCR), western blotting, immunohistochemistry (IHC) and immunofluorescence (IF), cell viability and proliferation assays, transwell assay, wound healing assay, luciferase assay, CUT&Tag assay, high-throughput data analyses, see Supplementary Information.

### Reporting summary

Further information on research design is available in the Nature Research Reporting Summary linked to this article.

### Supplementary information

Supplementary Information

Supplementary File of Western Blot

Reporting Summary

## Data Availability

All raw data have been deposited into the Research Data Deposit with the accession code RDDB2023459670 (http://www.researchdata.org.cn). Uncropped western blots are shown in Supplementary Information. The RNA-seq data generated in this study have been deposited in the GEO database with the accession code GSE245505. Other datasets generated during the current study are available from the corresponding author on reasonable request.
