# Supplementary material for: DNA methylation-activated full-length EMX1 facilitates metastasis through EMX1-EGFR-ERK axis in hepatocellular carcinoma
Source: Cell Death Dis. 2023 Nov 25;14(11):769. doi: 10.1038/s41419-023-06293-y (PMC10676392; doi:10.1038/s41419-023-06293-y)
Supplement: Supplementary file 1 — Supplementary Information [file 41419_2023_6293_MOESM1_ESM.pdf]

Supplementary Figure 1

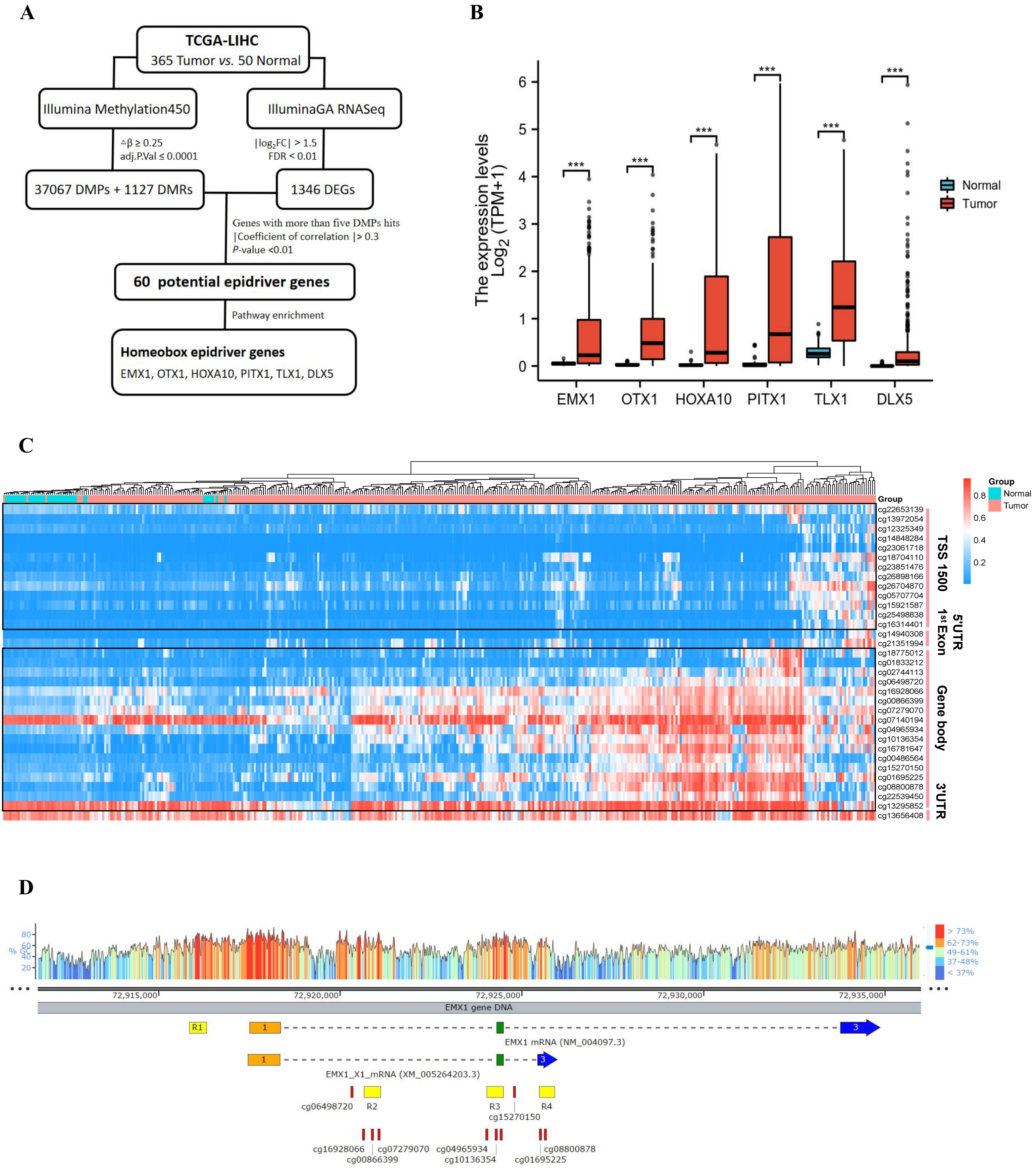

The figure continues on the next page

Supplementary Figure 1

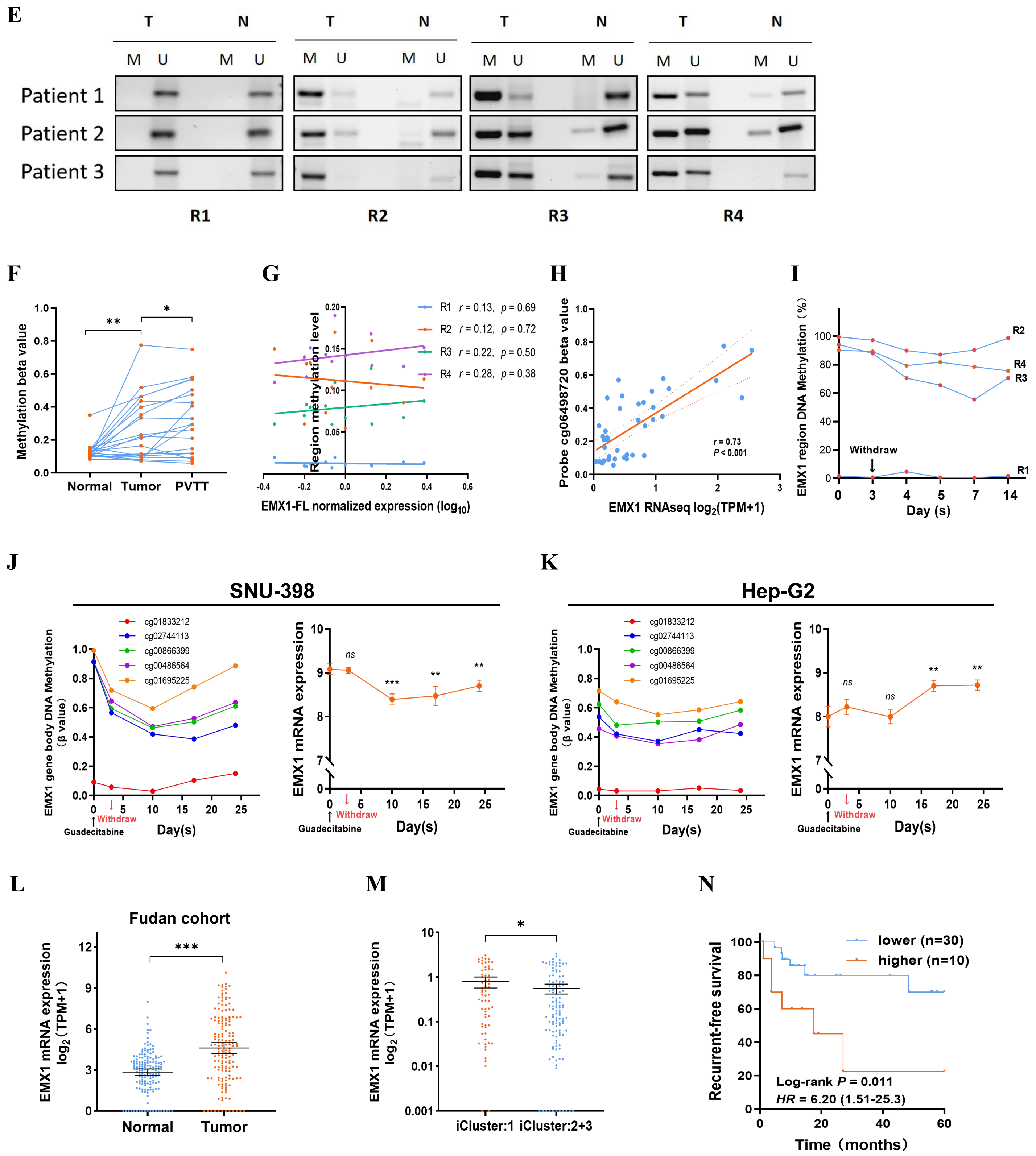

Supplementary Figure 2

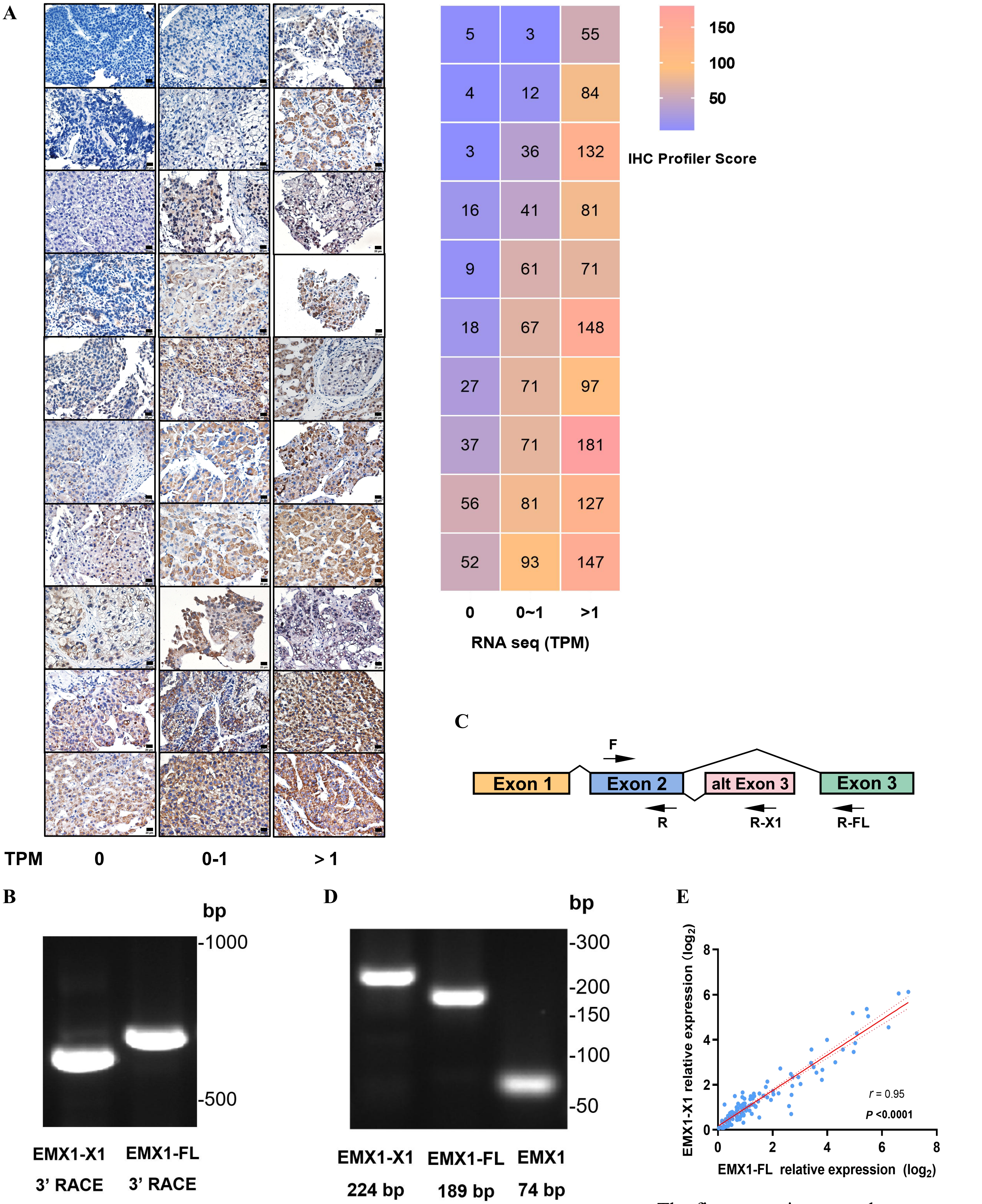

The figure continues on the next page

Supplementary Figure 2

F

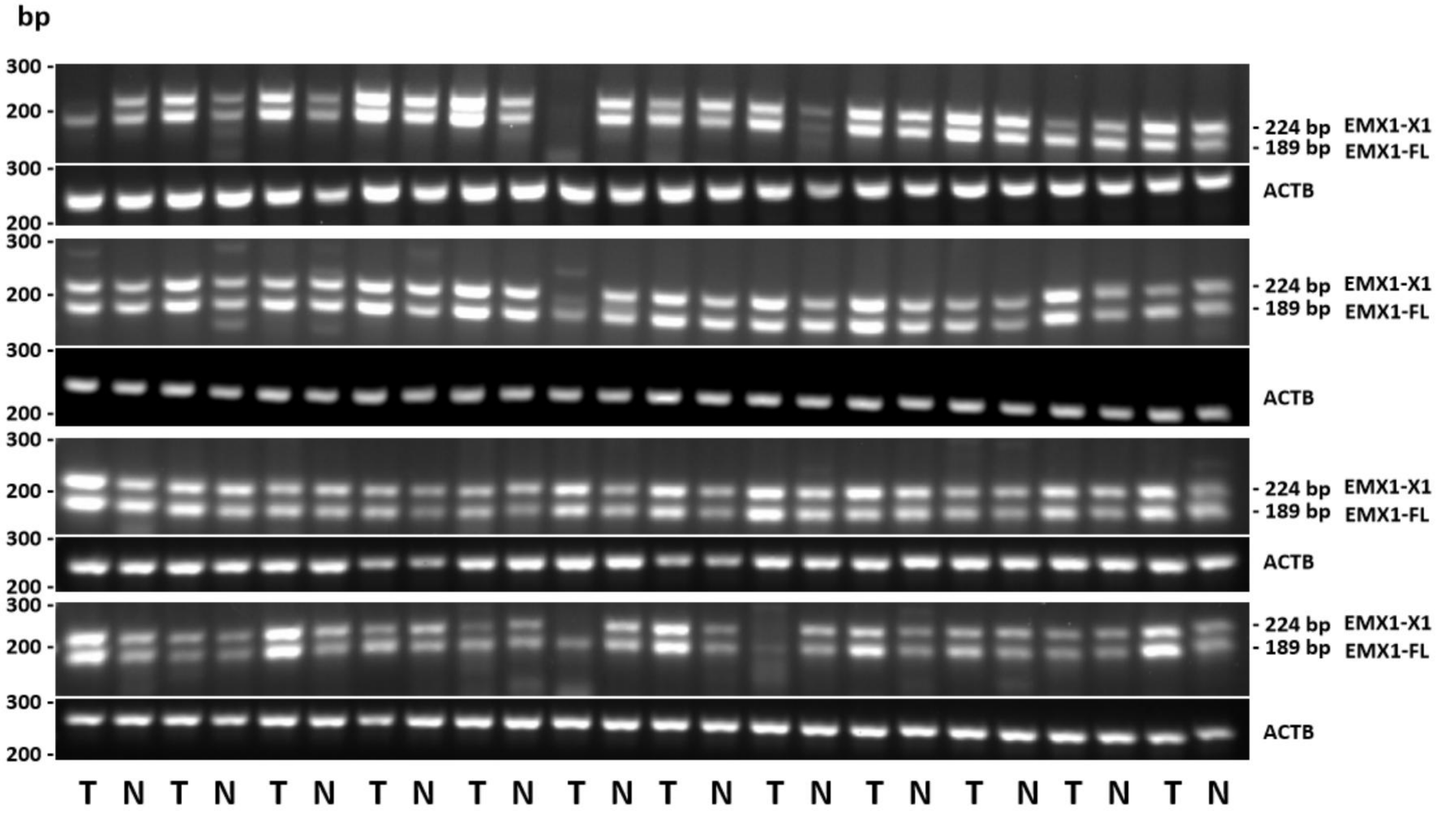

Supplementary Figure 3

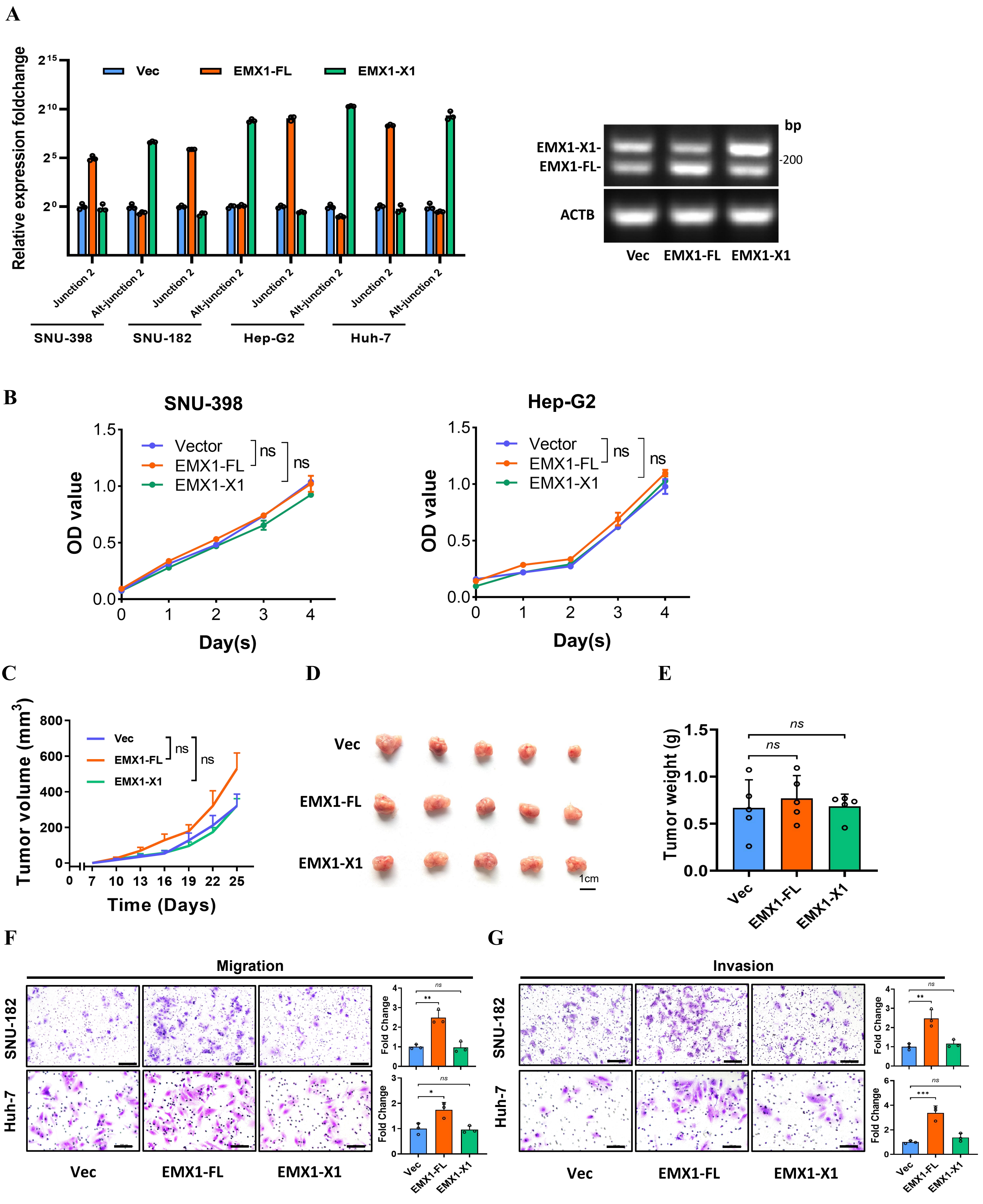

Supplementary Figure 4

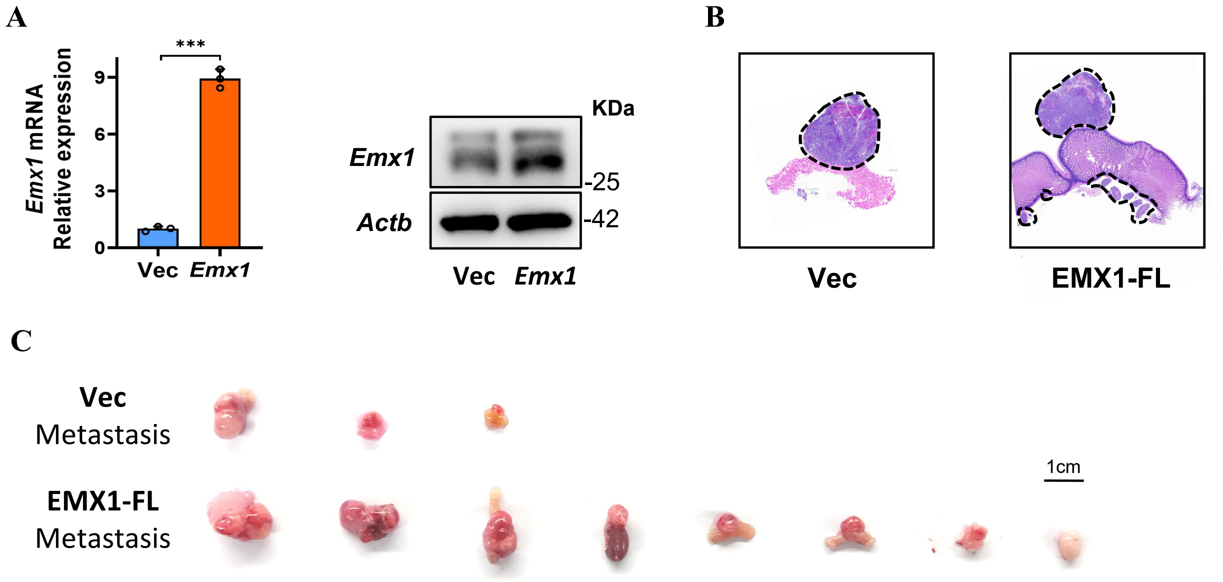

Supplementary Figure 5

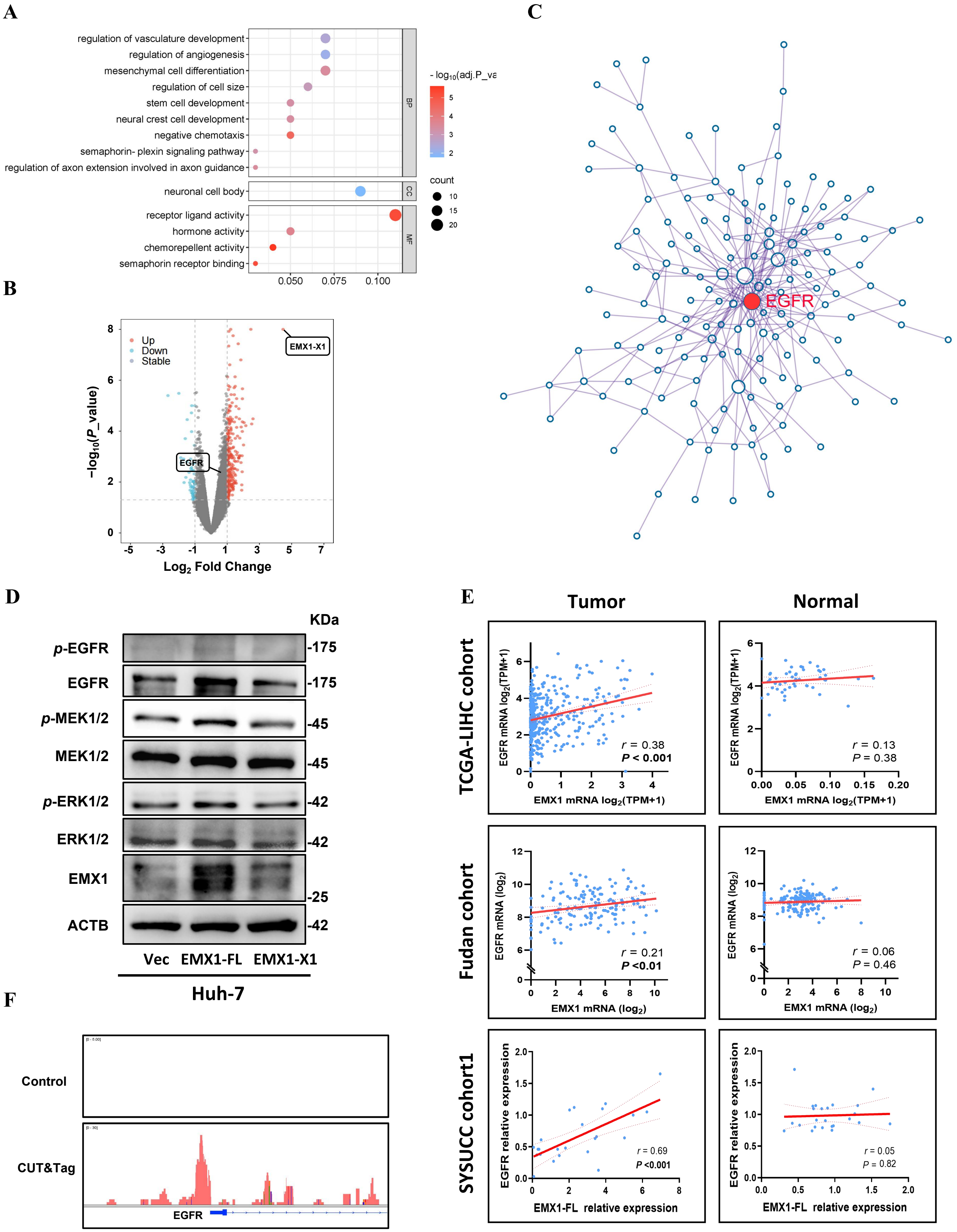

Supplementary Figure 6

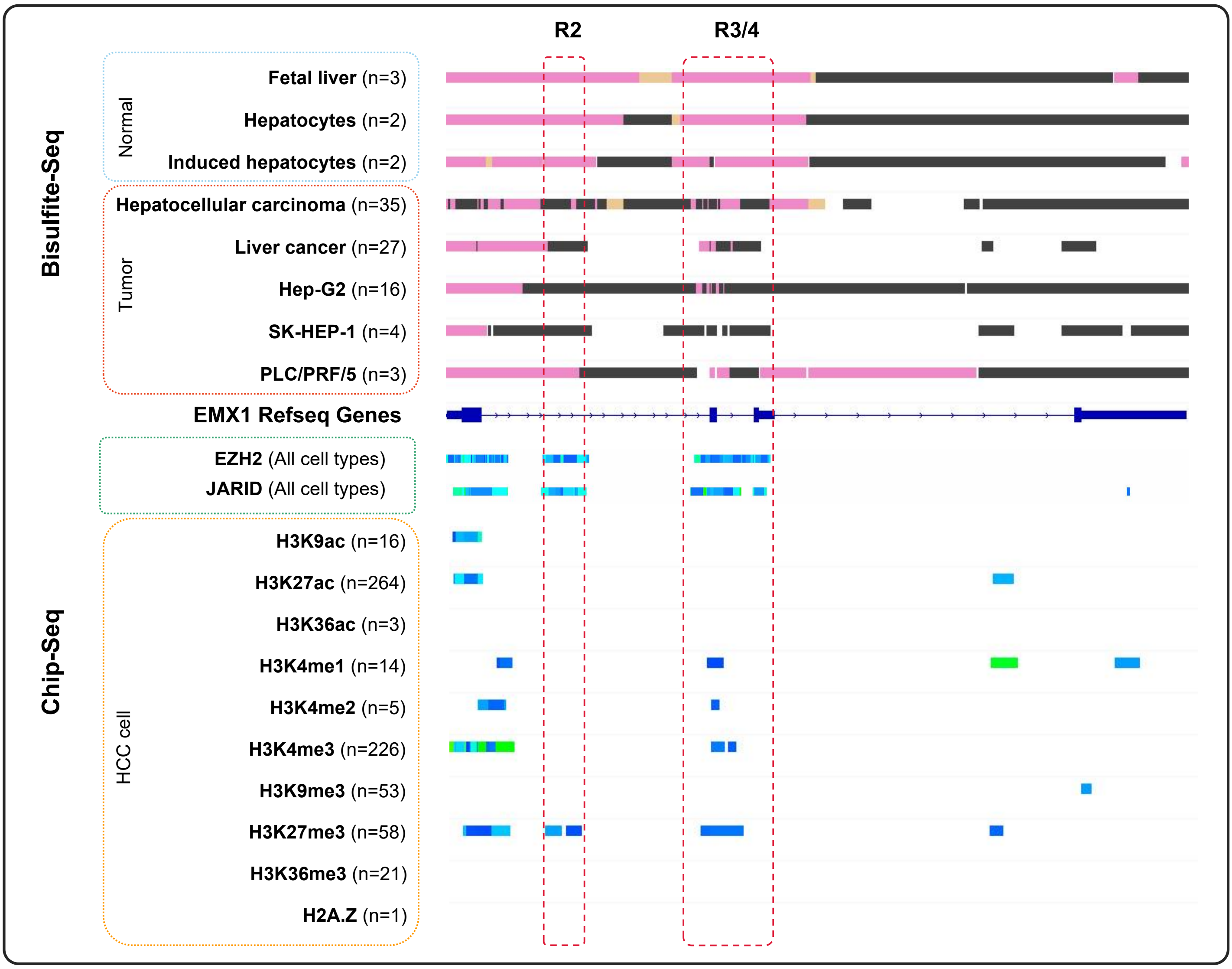

Supplementary Information

1. Supplementary Methods

1.1 Patients and public datasets

1.2 Bisulfite-next-generation sequencing PCR (BSP) and methylation-specific PCR (MSP)

1.3 3' Rapid amplification of cDNA ends (3'RACE)

1.4 Cell lines

1.5 Plasmids construct

1.6 Lentivirus production and infection

1.7 Transiently silencing and overexpression experiment

1.8 RNA extraction and quantitative real-time PCR (qRT-PCR) analysis

1.9 Western blot

1.10 Immunohistochemistry (IHC)

1.11 Immunofluorescence (IF) staining

1.12 Cell viability and proliferation assays

1.13 Transwell assay

1.14 Wound healing assay

1.15 Luciferase assay

1.16 CUT&Tag assay

1.17 High-throughput data bioinformatic analyses

2. Supplementary Materials

2.1 Supplementary tables

2.2 Supplementary figures

Abbreviations

ANLT, adjacent normal liver tissue; BSP, bisulfite sequencing PCR; CCK8, Cell Counting Kit-8; DEGs, differentially expressed genes; DMPs, differentially methylated probes; DMRs, differentially methylated regions; DNMTi, DNA methyltransferase inhibitors; EMX1-FL, EMX1 full-length; EMX1-X1, EMX1 alternative terminal exon splicing isoform; FBS, fetal bovine serum; HCC, hepatocellular carcinoma; IHC, immunohistochemistry; MSP, methylation-specific PCR; PCR, polymerase chain reaction; PVTT, portal vein tumor thrombus; qRT-PCR, quantitative real-time PCR; RNA-seq, RNA sequencing; *SD*, standard deviation; shRNA, short hairpin RNAs; siRNA, small interfering RNAs; SYSUCC, Sun Yat-sen University cancer center; TCGA-LIHC, The Cancer Genome Atlas of Liver Hepatocellular Carcinoma.

1. 1 Patients and public datasets

The TCGA-LIHC cohort was collected from http://www.cbioportal.org/ to analysis the genome-wide differentially methylated and expression gene. Details of the high-throughput data analyses are provided below. Fudan cohort was obtained from a previously published study[1]. SYSUCC cohort 1 contained sixty paired fresh-frozen HCC tumors and adjacent normal liver tissue (ANLT) specimens obtained from pathological diagnosis HCC patients who undergone surgery in Sun Yat-sen University Cancer Center (SYSUCC). Details of clinical characteristics are provided in the **Table S1**. SYSUCC cohort 2 (n=30, RNA-seq) was obtained by tumor biopsies from advanced HCC patients receiving oxaliplatin-based chemotherapy.

1.2 Bisulfite-next-generation sequencing PCR (BSP) and methylation-specific PCR (MSP)

According to CpG dinucleotide distribution characteristics of EMX1 (Gene ID: 2016; **Fig. S1E**), we designed four PCR regions to BSP to cover both the EMX1 promoter (R1) and gene body (R2, R3 and R4). The primers used in BSP are listed in **Table S2**.

The four target regions specific CpG dinucleotides sites are listed below:

BSP-Region 1 (with 44 CpG sites)

CAGGAGAGGGGGAAAATAAAGAGCCTGAGACACAAA**CG**AGAGGAAAAGACCA
TCACAGAAAGCTGGAAATCTC**CG**GAGAGGCCAG**CG**AGAACC**CGCG**CTCCCCA**CG**GA
TTCCATCATTCCTTC**CG**AAGG**CG**CCTCTG**CG**GTGTCTCAGC**CG**TGCCAGGCCC**CG**GG

GTTCCCAGGA**CGCG**GAGGAGTGCTGGGTG**CG**GC**CG**CCT**CG**CCTCCCCACCCCTGGC
CGCCCCTCCCCACCT**CG**CCCAAGGGGGC**CG**GAA**CG**G**CG**T**CG**G**CGCGCG**GGGGCTTT
T**CG**GAGCAGT**CG**AGTGGAAAATAGACTTTAACC**CG**CTTTGTGG**CG**GC**CG**GGG**CG**CC
CTGAG**CG**CTCTCCAAACCA**CG**GCTCC**CG**G**CG**CTCAGG**CG**GGC**CG**CTGCCAAGACC**C**
GGCCTGGAGTCCC**CG**CAGAGTTG**CGCG**G**CG**CA**CG**GACCC**CG**TGGCCTTGGGG**CG**TC
AGGAGGCCCAACCCAGATCT

BSP-Region 2 (with 44 CpG sites)

CTCTCTG**CG**CAG**CG**CTTGGG**CG**G**CGCG**GTCC**CG**G**CGCGCG**GGGAAG**CG**G**CG**T
CTC**CG**CTAAC**CG**AGG**CG**CTGGAAGGGGAAAAG**CG**AATG**CG**GAAT**CG**TCCAGGACTC
CGAAGGT**CG**GGGC**CG**CTCG**CG**AGCAC**CG**AAGGGGAGGAGC**CG**A**CG**AAGACCAGGA
GTGGGC**CG**CATTT**CG**GTACTGTTTCCC**CG**AGATCAGGAACTTTC**CG**GGTCTAGGAGCA
A**CG**CCTGGAGGGGGCTGTAGAGACCCAGCCCCC**CG**GGACC**CG**CAACTACAATGGGC
CGGAGCTTCTAAGGT**CG**CCTTTGTTCTGGCAGGAGGA**CG**GGGAATGAGGTTATCTC**C**
GC**CG**CCTGTCCTGCCTCTCCCTCTCCTAGCCCTAGGGCCCTC**CG**CCCAGC**CG**TC**CG**GC
CCTGAGCCCCTGGC**CG**G**CG**G**CG**GCCTCTCCAG**CG**AAGACTG**CG**GCT**CG**AAGACTGC
AGCT**CG**GACCC**CG**G

BSP-Region 3 (with 42 CpG sites)

CTTAACAGAGGGATCTGGAGAGCTGTTATTCCC**CGCG**TTCCCC**CGCG**GAGTGGCT
CT**CG**AGTG**CG**GGGAGGTGTTG**CG**GAGGGGAGTGGACTTAGGGAAGGGG**CG**GCAAAA
GGGCAAAGGGAGAAATGG**CG**TGTGTGTG**CG**TGTCAAGGAATGGAGAGGGCAGGG**CG**
CTTGGGAGCAGGG**CGCG**AGGCCAGGCTCTGTTGGGCCC**CG**GCTCA**CG**G**CG**CCCCTT
CTCTCTGTCTGTACCTG**CG**TGTGTTGC**CG**T**CG**G**CG**G**CG**GGGC**CG**CAGCCAG**CG**A**CG**T

GCCCCAGGA**CG**GGCTGCTTCTGCA**CG**GCCCCTT**CG**CA**CG**CAAGCCCAAG**CG**GATC**C**
GCA**CG**GCCTTCT**CG**CCCT**CG**CAGCTGCTG**CG**GCTGGAG**CGCG**CCTT**CG**AGAAGAAC
CACTA**CG**TGGTGGG**CG**C**CG**AG**CG**GAAGCAGCTGGC**CG**GCAGTCTCAGCCTCTC**CG**A
GA**CG**CAGG

BSP-Region 4 (with 23 CpG sites)

GCTGCCCTGGAGGTGGATTTCAGTCTCTG**CG**TGC**CG**GC**CG**GCTCCCAGAGTTG**C**
GAGAGGC**CG**GCTC**CGCG**GTCTCCCAGCTACCTCC**CG**GCTGACTTTTCACCTTC**CG**CT
CCCCTTTCCTCCTAGTCT**CG**ACCCTACTACCACCGTCCCCTCCCAAGTCC**CG**GGCA
GTGAGAAGATGCC**CG**GCATGGGGGGCAGC**CG**GAGCCTCCCTTTAGCAGCCAGAGTAG
GAAGGGGGCTTAGTGAGGGAGCCCAGACCCAAACTTCATCCGCAGCTTTCTT**CG**G**CG**
GACCTTACCCTCTCCTCCTTCAGTGGCATTTTGGCATCTATTGT**CG**TCATATCTGTCTGC
TGCCCCACTTAATCTACAAAT**CG**CTCA**CG**GGT**CG**GAGGCAGGACC**CG**TG**CG**TTTTCA
GATGTACTAGCTGGGCTGTTCTAACTGCAGGG

MSP of bisulfite-transformed DNA was carried out using specific methylated or unmethylated primers listed in **Table S3**.

1.3 3' Rapid amplification of cDNA ends (3'RACE)

RNA ligase-mediated rapid amplification of 3' cDNA ends was performed using a 3'RACE kit (Sangon Biotech, Shanghai, China) according to the manufacturer's instructions. In brief, total RNA was extracted from HCC tissues using TRIZOL/Chlorophorm/Phenol method. 2 μg of total RNA was used to generate cDNA with reverse transcriptase using a 3' adaptor primer. The first PCR was conducted

using a EMX1-specific forward primer and 3'RACE outer primer as reverse primer. Nested PCR was then performed using a nested EMX1-specific forward primer and a 3'RACE inner primer as reverse primer. The PCR product from the nested PCR was separated on a 2% agarose gel, and the gel extraction products were further subjected to sequencing. The EMX1-specific primers used for 3'RACE were listed in **Table S4**.

1.4 Cell lines

The SNU-398 and SNU-182 cells were purchased from Procell (Wuhan, China). The Hep-G2, Hep-3B, HEK293T and Hepa1-6 cells were purchased from the Cell Bank of the Chinese Academy of Sciences (Shanghai, China). Huh-7 was kindly provided by the National Cancer Centre Singapore (Singapore). All cell lines were characterized using short tandem repeat markers, and none were found in the International Cell Line Authentication Committee Database of Crosscontaminated or Misidentified Cell Lines. SNU-398 and SNU-182 cells were cultured in RPMI1640 (CORNING) with 10% fetal bovine serum (FBS, Sigma-Aldrich, USA) and 1% penicillin/streptomycin (Gibco, USA). Other cells were cultured in DMEM (CORNING) with 10% FBS and 1% penicillin/streptomycin. Cells were cultured in a humidified incubator with 5% CO_2 at 37℃. All cell lines were regularly tested for mycoplasma contamination and were confirmed to be mycoplasma-free.

1.5 Plasmids construct

For stable transfection experiments, human EMX1-FL and EMX1-X1

transcript-specific cDNA were cloned into the pEZ-Lv105 vector, and mouse *Emx1* cDNA was cloned into pReceiver-Lv181 (Flag-tag) vector, the short hairpin RNAs (shRNA) targeting EMX1-FL were constructed into psi-LVRU6GP vector (sh-EMX1-1, GCCTTCGAGAAGAACCACTAC; sh-EMX1-2, GAGTCCGAGCAGAAGAAGAAG). In particular, EMX1-FL N-terminal contains a potential alternative translation initial sites (ATG[34]), thus, we cloned EMX1-FL, EMX1-XI and EMX1$^{\triangle 1\text{-}33}$ into pEZ-M03 (GFP fusion tag) plasmids respectively for protein subcellular localization experiments. All these plasmids construction was supported by GeneCopoeia lnc. (Guangzhou, China).

1.6 Lentivirus production and infection

To produce lentivirus in HEK293T cells, the Lenti-Pac™ HIV expression packaging kit (GeneCopoeia, USA) was used. Virus-containing supernatant from HEK293T cells were collected and filtrated using 0.22 μm filters. The filtered supernatant was added to 70% confluent cells in the presence of 5-10 μg/ml polybrene (Sigma-Aldrich, USA). After 24 hours, the cells were incubated with a completely fresh medium containing the appropriate concentration of puromycin for screening stably transduced cells.

1.7 Transiently silencing and overexpression experiment

Lipofectamine3000 (ThermoFisher, USA) was used to transfect cells with small interfering RNAs (siRNAs) or plasmids following manufacturer’s instructions.

RiboBio (Guangzhou, China) provided the following EGFR siRNAs: siEGFR-1, GAGGAAATATGTACTACGA; siEGFR-2, GGAGCGAATTCCTTTGGAA; siEGFR-3, GCAGAGGAATTATGATCTT. The RNA knockdown effects were quantified by qRT-PCR, and the most efficient siRNA (siEGFR-2) was used to conduct further functional assays.

1.8 RNA extraction and quantitative real-time PCR (qRT-PCR) analysis

Total RNA was extracted from both cells and HCC tissues using the TRIZOL reagent (LifeTechnologies, USA) following the manufacturers' instructions. RNA quantity was measured using a NanoDrop 2000 spectrophotometer (ThermoFisher Scientific, USA). Total RNA was reverse transcribed to cDNA using ReverTra Ace qPCR RT Master Mix with gDNA remover (TOYOBO, Japan) according to the manufacturer's instructions. qRT-PCR analysis was conducted using THUNDERBIRD SYBR qPCR Mix (TOYOBO, Japan) and analyzed on a Bio-rad system (Bio-Rad CFX384 Touch, USA). The results were presented as fold change using the $2^{-\Delta\Delta CT}$ method with ACTB serving as an internal control. The primer sequences used in the qRT-PCR are listed in **Table S5**.

1.9 Western blotting

Whole-cell protein lysates were prepared using RIPA (KeyGEN, China). Protein concentration was quantified using the BCA assay (ThermoFisher Scientific, USA). Cell lysates were mixed with protein loading buffer before, boiled for 5 min at 95°C.

Equal amounts of protein lysates were loaded and run in 7.5-15% SDS-PAGE gel. Gels were transferred onto PVDF membrane (BIO-RAD, USA) at 250 mA for 90-150 min at 4°C. Membranes were then blocked with 5% non-fat milk in TBST at RT for 2 hours, incubated with primary antibodies overnight at 4°C and secondary antibodies at RT for 1 hour. Subsequently, they were scanned using the Bio-Rad imaging system. Primary antibodies such as EMX1, EGFR, *p*-EGFR, MEK, *p*-MEK, ERK1/2, *p*-ERK1/2 and ACTB were used in the study (details in **Table S6**). All experiments were performed for at least three biological repeats.

1.10 Immunohistochemistry (IHC)

Surgical resection and biospy samples without extensive necrosis or mouse tissues were fixed in 10% neutral formalin for 24 hours and then embedded in paraffin for sectioning. IHC staining was carried out as described previously. Briefly, liver sections of 4 μm thickness from each patient or mouse were rehydrated and incubated in 0.3% H_2O_2 to remove peroxidase activity, boiled with EDTA (pH 8.0) for antigen retrieval, and then blocked with 5% goat serum. The sections were then incubated with the primary antibody overnight at 4℃. After washing with PBS, tissue sections were incubated with HRP-conjugated secondary antibodies (ZSGB-BIO, Beijing, China) for 30 min at 37 ℃. The sections were then washed again with PBS and incubated with DAB solution. The slides were counterstained with hematoxylin, sealed and observed under a microscopy (Olympus, Japan). Quantification of percent positive regions was performed using the IHC profiler plug-in for ImageJ[2].

1.11 Immunofluorescence (IF) staining

SNU-398 cells were seeded on 96-well plates (CellCarrier^{TM}-96 ultra, PerkinElmer) and transfected with empty GFP vector, or constructs expressing GFP fusion EMX1-FL, EMX1-X1, EMX1$^{\triangle 1\text{-}33}$. After 48h, cells were fixed in 4% paraformaldehyde at RT for 20 min, permeabilized in 0.1% Triton X-100 in PBS at RT for 5 min and then incubated in 1% BSA in PBS with 0.1% Phalloidin-iFluor 594 antibody (Abcam, ab176757) at RT for 1h. Afterwards, the cells were stained with Hochest, and were visualized with high-content imaging at 60x resolution with the Operetta CLS High-content Imaging System (PerkinElmer, USA).

1.12 Cell viability and proliferation assays

2000 cells were seeded into each well of a 96-well plates with a complete medium. Cell viability and proliferation were evaluated using the Cell Counting Kit-8 (CCK8, Dojindo, Japan) at 4, 24, 48, 72 and 96 hours after seeding. The optical density was then detected by a microplate reader at 450nm and 600nm (Bio-Tek Epoch, USA). Readouts (450nm - 600nm) were presented as growth curves.

1.13 Transwell assay

Cells were serum-starved for 24 hours before being plated into Falcon cell culture inserts (CORNING) for migration assay or premade 10% matrigel inserts for invasion assay. Cells were resuspended in serum-free medium and migrated/invaded into lower

culture medium with 20% FBS. After the non-migrated/invaded cells were removed, the inserts were washed in PBS, fixed in 100% methanol, air-dried, stained with 0.5% crystal violet, washed in distilled H_2O, air-dried, and imaged. The migrated/invaded cells were then counted using ImageJ (NIH). Fold change was calculated by the number of migrated/invaded cells relative to the control condition. For SNU-398, SNU-182 and Huh-7 cells, 5×10^4 were seeded and incubated 24-48 hours for migration and 48 hours for invasion assay. For the typical epithelial cell line Hep-G2 migration and invasion assays, 5-10×10^4 cells were seeded and incubated for 48-72 hours prior to fixation and quantification. At least three independent experiments were performed.

1.14 Wound healing assay

SNU-398 or Hep-G2 were serum-starved for 24 hours before being seeded at a density of 5×10^4 cells/well into Culture-Insert (ibidi, Germany). After 24 hours, the Culture-Insert was gently removed using sterile tweezers, and the culture medium was filled with 2.5% FBS. Phase-contrast images were captured at the initial time and after 48 hours for SNU-398 or 96h for Hep-G2. All experiments were performed in triplicate.

1.15 Luciferase assay

The luciferase reporter assay was performed according to the standard protocol of the Dual-Luciferase Reporter Assay system (Promega, USA). Briefly, the EGFR

full-length promoter (-1500bp from transcription start site), truncating mutations and point mutation promoter were cloned into pEZX-FR01 luciferase reporter plasmids (GeneCopoeia, Guangzhou, China) and transfected into SNU-398 cells using lipo3000 (Invitrogen, USA). Cells were collected and lysed by lysis buffer 24 hours later, after which luciferase activity was determined using GloMax Navigator (Promega, USA). Renilla luciferase activity was normalized against firefly luciferase activity.

1.16 CUT&Tag assay

The CUT&Tag assay was performed according to the manufacturer's instruction of NovoNGS CUT&Tag High-Sensitivity Kit V2.0 (Novoprotein, Shanghai, China). Briefly, 10^6 cells of EMX1-FL overexpressed SNU-398 per sample were harvested and washed with buffer. ConA magnetic bead-bound cells were resuspended in 50 μL precooled Primary Antibody Buffer containing the primary antibody (EMX1, Sigma-Aldrich). Rabbit IgG was used as the control. After removing the primary antibody, the diluted secondary antibody and cells were incubated at RT for 1 hour. ChiTag™ 2.0 Transposome was used to resuspend the cells, and the incubation was performed at RT for 1 hour. After cells were incubated in Tagmentation Buffer on a rotating platform at 37 °C for 1 hour, then the DNA was extracted for library construction and sequencing was performed on the Illumina NovaSeq 6000 platform conducted by NuoBiotech (Guangzhou, China). Paired-end reads were mapped to a reference genome using Bowtie2[3]. Peaks were visualized using Integrative

Genomics Viewer. EGFR promoter enrichment degree was examined by qRT-PCR. Primers used are listed in **Table S5**.

1.17 High-throughput data bioinformatic analyses

Aberrant methylation loci are usually found around the promoter but do not always necessarily contribute to gene silencing or activity. For biological exploration purposes, we expanded the analysis of all HumanMethylation450 BeadChip CpG islands, including TSS1500, TSS200, 5'UTR (untranslated region), 1st Exon, gene body and 3'UTR. For TCGA-LIHC-methy profile profile (n=368), the remaining probes after downloading and quality control were utilized to identify differentially methylated probes (DMPs) and differentially methylated regions (DMRs) between tumor and normal using the ChAMP R pipeline[4]. An adj.$P < 0.0001$ and $|\beta_{tumor}-\beta_{normal}| > 0.25$ was considered to be significant. All 36 probes of the EMX1 gene from TSS1500 to the 3'UTR were collected, and its DNA methylation genomic location, expression and clinical data were visualized using MEXPRESS[5,6]. For TCGA-LIHC RNA-seq profile (n=371), differentially expressed genes (DEGs) between tumor and normal were calculated by R package DEseq2[7]. FDR was calculated from multiple testing corrections of raw *P* value via the Benjamini and Hochberg method. Finally, $|\log_2(\text{fold change})| > 1.5$ and FDR < 0.01 were set as the threshold for DEGs. Sixty genes were extracted as potential HCC epidrivers following two criteria: genes with more than five DMPs hits; at least one methylated probe with the gene mRNA expression |confficient of correlation| > 0.3 and the *P*-value <0.01. To

explore the biology of the sixty potential HCC epidriver genes or DEGs, we used DAVID for the gene ontology analysis[8]. The $P < 0.01$ and $FDR < 0.25$ was considered statistical significance. The top highly enriched terms are shown.

The correlation between EMX1 mRNA transcripts and DNA methylation status at the EMX1 loci was determined using Pearson's correlation analysis and linear regression. Pearson's correlation coefficient (r) was calculated for EMX1-FL mRNA expression and methylation status, including entire regions and individual CpG sites. The strength of correlation was considered as follows: weak, $0 < r < 0.4$; moderate, $0.4 \leqslant r < 0.8$; or strong, $0.8 \leqslant r < 1.0$. A negative r-value indicates an inverse/negative correlation, whereas a positive r-value indicates a direct/positive correlation.

For SNU-398 EMX1 transcript-specific overexpression cell lines, RNA isolation and RNA-seq were performed following standard protocols as previously reported. In brief, RNA was extracted using TRIZOL/Chlorophorm/Phenol method. RNA-seq was performed on the Illumina NovaSeq 6000 platform conducted by Novogen (Beijing, China). Raw data of Fastq format were processed with Fastp for read trimming and read-level quality control. Reads were then aligned against the human reference genome using STAR[9] under default parameters. The "rsem-calculate-expression" script of RSEM[10] was used to count the reads numbers mapped to each gene. Transcript per million of each gene was calculated based on the length of the gene and reads count mapped to this gene. $|\log_2(\text{fold change})| \geqslant 1$ and p value< 0.05 were set as the threshold for DEGs. Gene Ontology analysis upon DEGs were performed to explore the potential downstream pathways.

2.1 Supplementary tables

Table S1. clinical characteristics of SYSUCC cohort 1 (n=60).

Variables	N (%)	Variables	N (%)
Age	52.3±10.0	Tumor size (cm)	5.6±3.1
＞50	37 (61.7)	＞5	25 (41.7)
≤50	23 (38.3)	≤5	35 (58.3)
Gender		Tumor number	
Male	52 (86.7)	Multiple	12 (20.0)
Female	8 (13.3)	Single	48 (80.0)
HBsAg		PVTT	
Positive	56 (93.3)	Yes	3 (5.0)
Negative	4 (6.7)	No	57 (95.0)
Cirrhosis		Microvascular invasion	
Yes	34 (56.7)	Yes	18 (30.0)
No	26 (43.3)	No	42 (70.0)
AFP (ng/ml)		Tumor differentiation	
＞400	22 (36.7)	Well	26 (43.3)
≤400	38 (63.3)	Poor	34 (56.7)

Abbreviation: HBsAg, hepatitis B surface antigen; AFP, alpha fetoprotein; PVTT, portal vein tumor thrombus.

Table S2. Primers used in bisulfite sequencing PCR (BSP).

BSP primers	Sequences	Product (bp)
BSP-R1-F	TAGGAGAGGGGGAAAATAAAGAG	465
BSP-R1-R	AAATCTAAATTAAACCTCCTAACRC	
BSP-R2-F	TTTTTTGYGTAGYGTTTGG	462
BSP-R2-R	CCRAAATCCRAACTACAATCTT	
BSP-R3-F	TTTAATAGAGGGATTTGGAGAGTTG	456
BSP-R3-R	CCTACRTCTCRAAAAAACTAAAACTAC	
BSP-R4-F	GTTGTTTTGGAGGTGGATTTTAGT	432
BSP-R4-R	CCCTACAATTAAAACAACCCAACT	

Table S3. Primers used in methylation-specific PCR (MSP).

MSP primers	Sequences	Product (bp)
MSP-R1-M-F	TATTTCGTTTAAGGGGGTCG	165
MSP-R1-M-R	CCAAACCGAATCTTAACAACG	
MSP-R1-U-F	TTTGTTTAAGGGGGTTGGAA	157
MSP-R1-U-R	CCAAATCTTAACAACAACCC	
MSP-R2-M-F	GTTTAGGATTTCGAAGGTCGGG	178
MSP-R2-M-R	CCATTATAATTACGAATCCCGAAA	
MSP-R2-U-F	TTAGGATTTTGAAGGTTGGGG	176
MSP-R2-U-R	CCATTATAATTACAAATCCCAAAA	

MSP-R3-M-F	GTAGTTAGCGACGTGTTTTAGGAC	166
MSP-R3-M-R	TACCGACCAACTACTTCCG	
MSP-R3-U-F	TGATGTGTTTTAGGATGGG	166
MSP-R3-U-R	ACTAAAACTACCAACCAACTACTTCCA	
MSP-R4-M-F	GAGTGGGGTTTAGCGATT	213
MSP-R4-M-R	GAACATCTTCTCACTACCCGAAA	
MSP-R4-U-F	TGGAGTGGGGTTTAGTGATT	219
MSP-R4-U-R	TACCAAACATCTTCTCACTACCCA	

Table S4. Primers used in 3'RACE.

3'RACE primers	Sequences
3' adaptor Primer	GCTGTCAACGATACGCTACGTAACGGCAT GACAGTGTTTTTTTTTTTTTTTTTT
3' RACE Outer Primer	GCTGTCAACGATACGCTACGTAAC
3' RACE Inner Primer	GCTACGTAACGGCATGACAGTG
3' RACE EMX1-FL Outer	CAGGACGGGCTGCTTCTG
3' RACE EMX1-FL Inner	GCGCCTTCGAGAAGAACCA
3' RACE EMX1-X1 Outer	GCGCCTTCGAGAAGAACCA
3' RACE EMX1-X1 Inner	CAGGTCCGACGTGTTGGAGT
3' RACE Control F1	CCTGAGCTGAACGGGAAGCTCACTG
3' RACE Control F2	CCTTCCGTGTCCCCACTGCCAA

Table S5. Primers used in qRT-PCR.

qRT-PCR primers	Sequences
EMX1-F	GCGCCTTCGAGAAGAACCA
EMX1-all-R	CTCGGAGAGGCTGAGACTGC
EMX1-FL-R	ACCGGTTGATGTGATGGGAG
EMX1-X1-R	GAAAAGTCAGCCGGGAGGTAG
Emx1-F	CTCACTCTTTCTTCAGCGCC
Emx1-R	TTCTTCTCAAAGGCTCGCTCC
ACTB-F	CATGTACGTTGCTATCCAGGC
ACTB-R	CTCCTTAATGTCACGCACGAT
EGFR-F	AGGCACGAGTAACAAGCTCAC
EGFR-R	ATGAGGACATAACCAGCCACC
EGFR-promoter-F	GGTGGGGACCCGAATAAAGG
EGFR-promoter-R	TTTAGACAGACTGGCCGAGC
EGFR-promoter-control-F	AGGCATGCTCCTGGTACAAA
EGFR-promoter-control-R	ACTAGGTAGCTCGGGGTCAG

Table S6. Antibodies used the study.

Antibody	Source	Cat. #	Application and dilution
Phalloidin-iFluor 594	Abcam	ab176757	IF 1:1000
EMX1	Sigma-Aldrich	HPA006421	WB 1:1000

			IHC 1:200
EMX1	Thermofisher	PA5-35373	WB 1:1000
EGFR	CST	4276	WB 1:1000
p-EGFR (Y1068)	CST	3777	WB 1:1000
ERK1/2	CST	9102	WB 1:1000
p-ERK1/2	CST	4370	WB 1:1000
MEK	CST	9126	WB 1:1000
p-MEK	CST	9154	WB 1:1000
ACTB	CST	3700	WB 1:1000
Anti-Rabbit Secondary antibodies	CST	7074	WB 1:5000
Anti-Mouse Secondary antibodies	CST	7076	WB 1:5000

CST, Cell Signaling Technology.

2.2 Supplementary figure legend

Supplementary Figure 1. EMX1 is an epidriver and its gene body hypermethylation enhanced EMX1 expression in HCC. (**A**) The flowchart for joint analysis of TCGA-LIHC cohort DNA methylome and transcriptome. DMPs, differentially methylated probes; DMRs, differentially methylated regions; DEGs, differentially expressed genes. (**B**) The RNA expressions level of six Homeobox genes (EMX1, OTX1, HOXA10, PITX1, TLX1 and DLX5) in HCC tumors and

normal tissues in the TCGA-LIHC cohort. $^{***}P < 0.001$. (**C**) The status of EMX1 methylation probes in tumor and normal tissues of TCGA-LIHC cohort. (**D**) The CG content of the EMX1 gene and the top ten DMPs loci (cg06498720, cg16928066, cg00866399, cg07279070, cg04965934, cg10136354, cg16781647, cg15270150, cg01695225 and cg08800878; adj.$P<0.0001$ and $|\beta_{tumor}-\beta_{normal}| > 0.25$). (**E**) MSP assay detected the methylation status of EMX1 gene from R1 to R4 from HCC patient tumor tissues and ANLT (M, methylated; U, unmethylated). (**F**) The DNA methylation status at probe cg06498720 (locate in R2) was examined in 60 matched samples, including HCC primary tumor, PVTT and ANLT from 20 HCC patients (GSE77269). $^{*}P < 0.05$; $^{**}P < 0.01$. (**G**) Scatterplot of the correlation between EMX1 regions methylation status and mRNA expression (normalized and $\log_{10}$ transformed) in ANLT (n=12). (**H**) Scatterplot of the correlation between EMX1 cg06498720 methylation status and mRNA expression (RNA-seq) in HCC primary tumor and PVTT (n=40, GSE77269). (**I**) Quantification of MSP assay of SNU-398 treated with Decitabine. (**J-K**) The EMX1 gene body methylation status (GSE105066, left panel) and mRNA expression (GSE105065, right panel) in SNU-398 (**J**) and Hep-G2 (**K**) treated with Guadecitabine. Data shown represent the means ($\pm$*SD*) of four biological replicates. *ns*, no significant; $^{**}P < 0.01$; $^{***}P < 0.001$. (**L-M**) The EMX1 total mRNA expression was elevated in HCC tumor tissues derived from the Fudan cohort (**L**) and TCGA-LIHC iCluster1 (**M**). The mRNA expression data was shown to represent the means ($\pm$ 95% confidence interval). $^{***}P < 0.001$. (**N**) Kaplan-Meier analysis of recurrent-free survival in early stage HCC (initial surgery patients with single tumor,

n=40) from SYSUCC cohort 1 based on EMX1 gene expression.

Supplementary Figure 2. HCC exploits an EMX1 mRNA terminal exon splicing event and regulates its nucleus location. (**A**) Left panel: IHC with anti-EMX1 was performed in HCC biospy samples indicated different EMX1 protein expression pattern and subcellular location. Scale bars, 20 μm. Right panel: Quantification of the IHC positive score using ImageJ. (**B**) 3'RACE PCR showing the two alternative third exon lengths in HCC tumor cDNA. (**C**) The transcript-specific intron-spanning primers were designed for qRT-PCR (primer F+R: 74 bp; primer F+R-FL: 189 bp; primer F+R-X1: 224 bp). (**D**) Representative image of agarose gel electrophoresis separated transcript-specific EMX1 PCR products. (**E**) Scatterplot of the correlation between EMX1-FL mRNA expression and EMX1-X1 mRNA expression in HCC tumor (SYSUCC cohort 1, n=60). (**F**) The PCR products of two EMX1 transcripts were separated through 3% agarose gel electrophoresis and visualized with GelRed in 48 paired HCC tumor and ANLT samples.

Supplementary Figure 3. EMX1-FL but not its alternative splicing isoform enhances HCC migration and invasion ability *in vitro*. (**A**) Forced expression of transcript-specific EMX1 in HCC cell lines (including SNU-398, SNU-182, Hep-G2 and Huh-7) and validated by qRT-PCR (left panel) and a representative PCR sizing gel (right panel, EMX1-FL was amplified with primers spanning intron 2 as 189bp; EMX1-X1 was amplified with primers spanning alternative intron 2 as 224bp). (**B**)

CCK8 assays were conducted in ectopic expression of transcript-specific EMX1 of both SNU-398 (left panel) and Hep-G2 (right panel). Error bars represent the mean ± *SD* of three biological replicates. *ns*, no significant. (**C-E**) Ectopic expression of transcript-specific EMX1 in SNU-398 did not promoted the tumour growth in subcutaneous xenograft model as shown in tumor proliferation curves (**C**), error bars represent the mean ± *SEM* of five biological replicates; representative pictures of subcutaneous tumor nodules (**D**) and quantification of tumor nodules weight (**E**) (n=5), error bars represent the mean ± *SD* of five biological replicates. *ns*, no significant. (**F-G**) Representative images and quantification of *in vitro* migration (**F**) and invasion (**G**) assays in SNU-182 (upper panel) and Huh-7 (lower panel) cells stably transfected with transcript-specific EMX1 or vector control. Scale bar, 100μm.

Supplementary Figure 4. EMX1-FL enhances HCC metastasis *in vivo*. (**A**) Forced expression of *Emx1* in Hepa1-6 using *musculus Emx1* overexpression plasmid and validated by qRT-PCR (left panel) and western blotting (right panel). $^{***}P < 0.001$. (**B-C**) The orthotopic xenograft HCC model extrahepatic metastasis nodules. Representative HE staining images (**B**) and gross images (**C**). Black dotted box indicates extrahepatic metastatic nodules. Error bars represent the mean ± *SD*. $^{***}P < 0.001$.

Supplementary Figure 5. EMX1-FL promotes liver cancer EGFR signaling. (A-B) The DEGs of SNU-398 EMX1-X1 overexpression were enriched by Gene Ontology

analysis (**A**) and shown as a volcano plot (**B**). (**C**) The protein-protein interaction of SNU-398 EMX1-FL overexpressing DEGs. EGFR was a hub gene and highlighted with a red circle. (**D**) Ectopic expression of EMX1-FL, but not the EMX1-X1, increased the total EGFR protein levels and activated EGFR-ERK signaling in Huh-7. (**E**) Scatterplots of the expression correlation in tumor (left panel) or ANLT (right panel) between EMX1 mRNA and EGFR mRNA in the TCGA-LIHC cohort, Fudan cohort and SYSUCC cohort 1 (n=24). (**F**) The EMX1-FL CUT&Tag sequencing peaks located in EGFR promoter were visualized using Integrative Genomics Viewer.

Supplementary Figure 6. The integrate data of bisulfite sequencing (upper panel) and chromatin immunoprecipitation sequencing (lower panel) in liver or HCC tissues/cells associated with EMX1 gene loci (data collected from http://chip-atlas.org/).

References

1Gao Q, Zhu H, Dong L, Shi W, Chen R, Song Z et al. Integrated Proteogenomic Characterization of HBV-Related Hepatocellular Carcinoma. CELL. 2019;179(2):561-77. 'doi:'10.1016/j.cell.2019.08.052.

2Varghese F, Bukhari AB, Malhotra R, De A. IHC Profiler: an open source plugin for the quantitative evaluation and automated scoring of immunohistochemistry images of human tissue samples. PLOS ONE. 2014;9(5):e96801. 'doi:'10.1371/journal.pone.0096801.

3Langmead B, Salzberg SL. Fast gapped-read alignment with Bowtie 2. NAT METHODS.

2012;9(4):357-9. 'doi:'10.1038/nmeth.1923.

4Morris TJ, Butcher LM, Feber A, Teschendorff AE, Chakravarthy AR, Wojdacz TK et al. ChAMP: 450k Chip Analysis Methylation Pipeline. BIOINFORMATICS. 2014;30(3):428-30. 'doi:'10.1093/bioinformatics/btt684.

5Koch A, De Meyer T, Jeschke J, Van Criekinge W. MEXPRESS: visualizing expression, DNA methylation and clinical TCGA data. BMC GENOMICS. 2015;16(1):636. 'doi:'10.1186/s12864-015-1847-z.

6Koch A, Jeschke J, Van Criekinge W, van Engeland M, De Meyer T. MEXPRESS update 2019. NUCLEIC ACIDS RES. 2019;47(W1):W561-5. 'doi:'10.1093/nar/gkz445.

7Love MI, Huber W, Anders S. Moderated estimation of fold change and dispersion for RNA-seq data with DESeq2. GENOME BIOL. 2014;15(12):550. 'doi:'10.1186/s13059-014-0550-8.

8Sherman BT, Hao M, Qiu J, Jiao X, Baseler MW, Lane HC et al. DAVID: a web server for functional enrichment analysis and functional annotation of gene lists (2021 update). NUCLEIC ACIDS RES. 2022;50(W1):W216-21. 'doi:'10.1093/nar/gkac194.

9Dobin A, Davis CA, Schlesinger F, Drenkow J, Zaleski C, Jha S et al. STAR: ultrafast universal RNA-seq aligner. BIOINFORMATICS. 2013;29(1):15-21. 'doi:'10.1093/bioinformatics/bts635.

10Li B, Dewey CN. RSEM: accurate transcript quantification from RNA-Seq data with or without a reference genome. BMC BIOINFORMATICS. 2011;12:323. 'doi:'10.1186/1471-2105-12-323.
