# Supplementary material for: DNA methylation-activated full-length EMX1 facilitates metastasis through EMX1-EGFR-ERK axis in hepatocellular carcinoma
Source: Cell Death Dis. 2023 Nov 25;14(11):769. doi: 10.1038/s41419-023-06293-y (PMC10676392; doi:10.1038/s41419-023-06293-y)

Uncropped gels for Western blot

Figure 2A

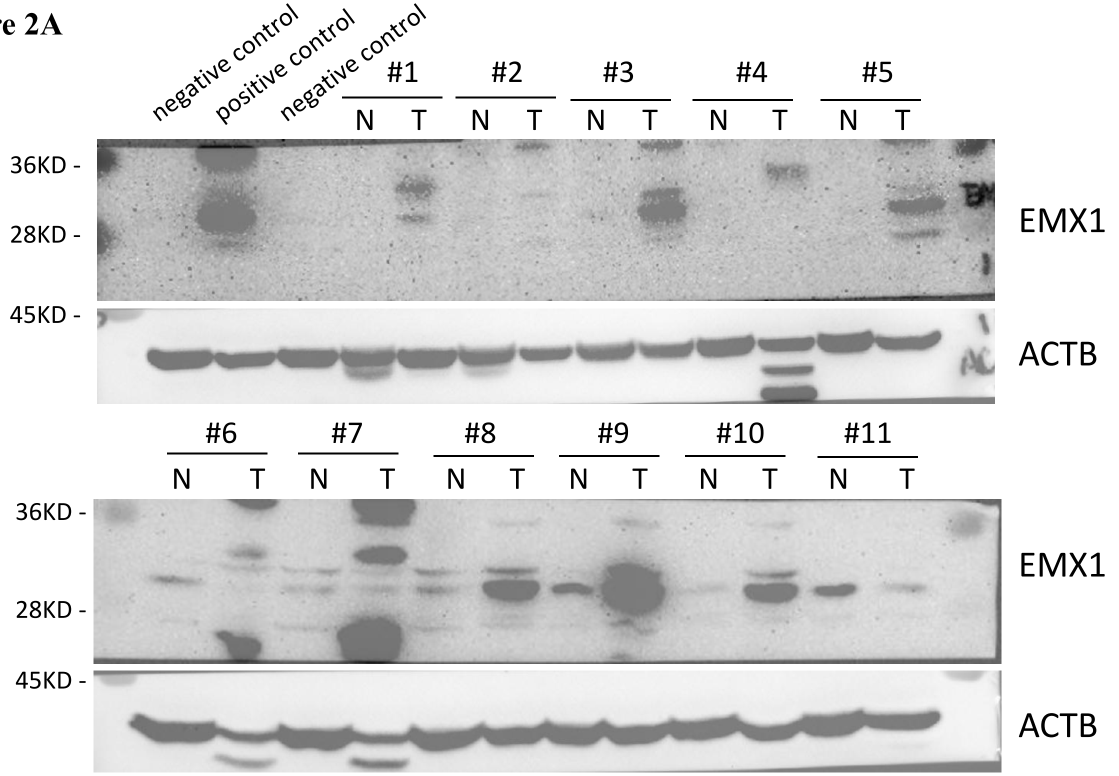

Figure 3C

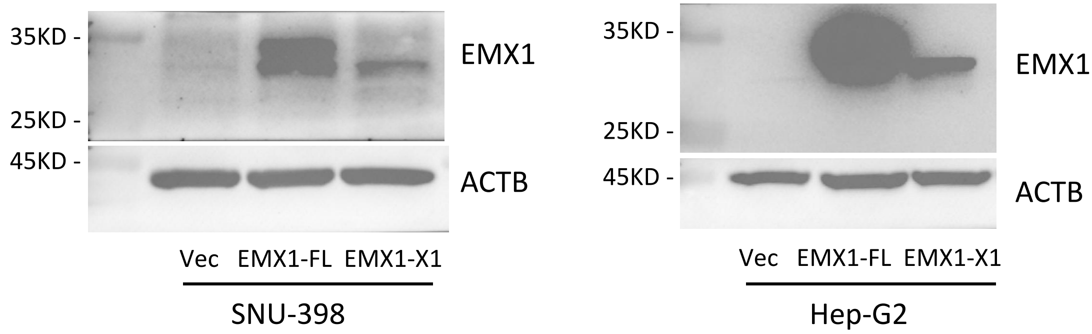

Figure 3G

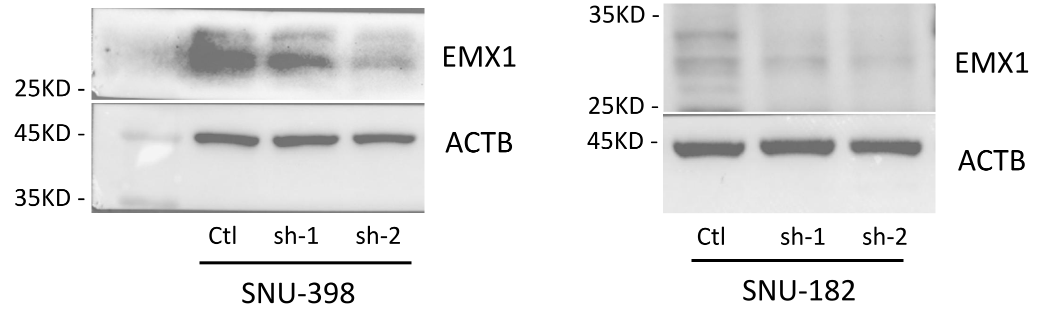

Figure 5E

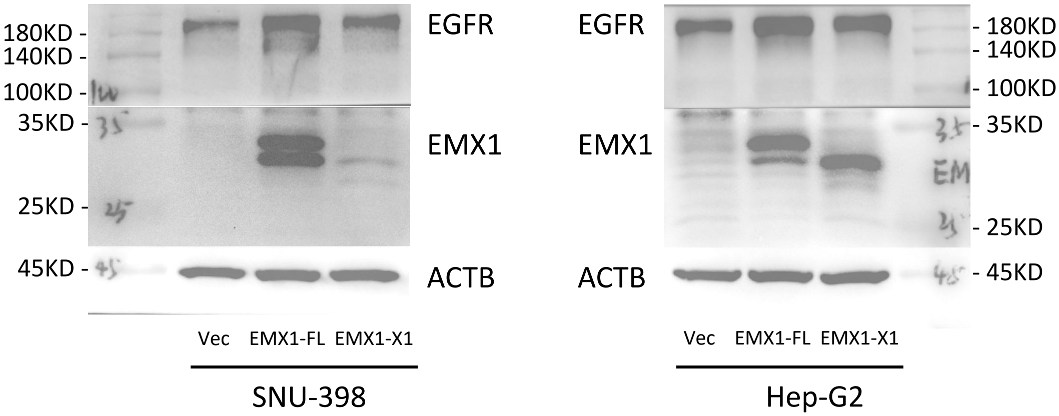

Figure 5F

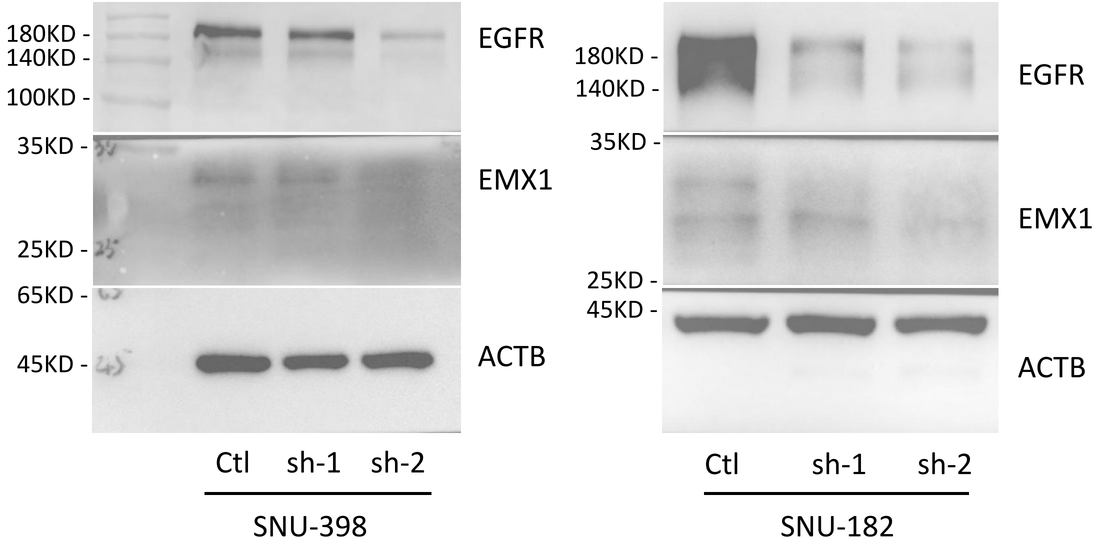

Figure 5H

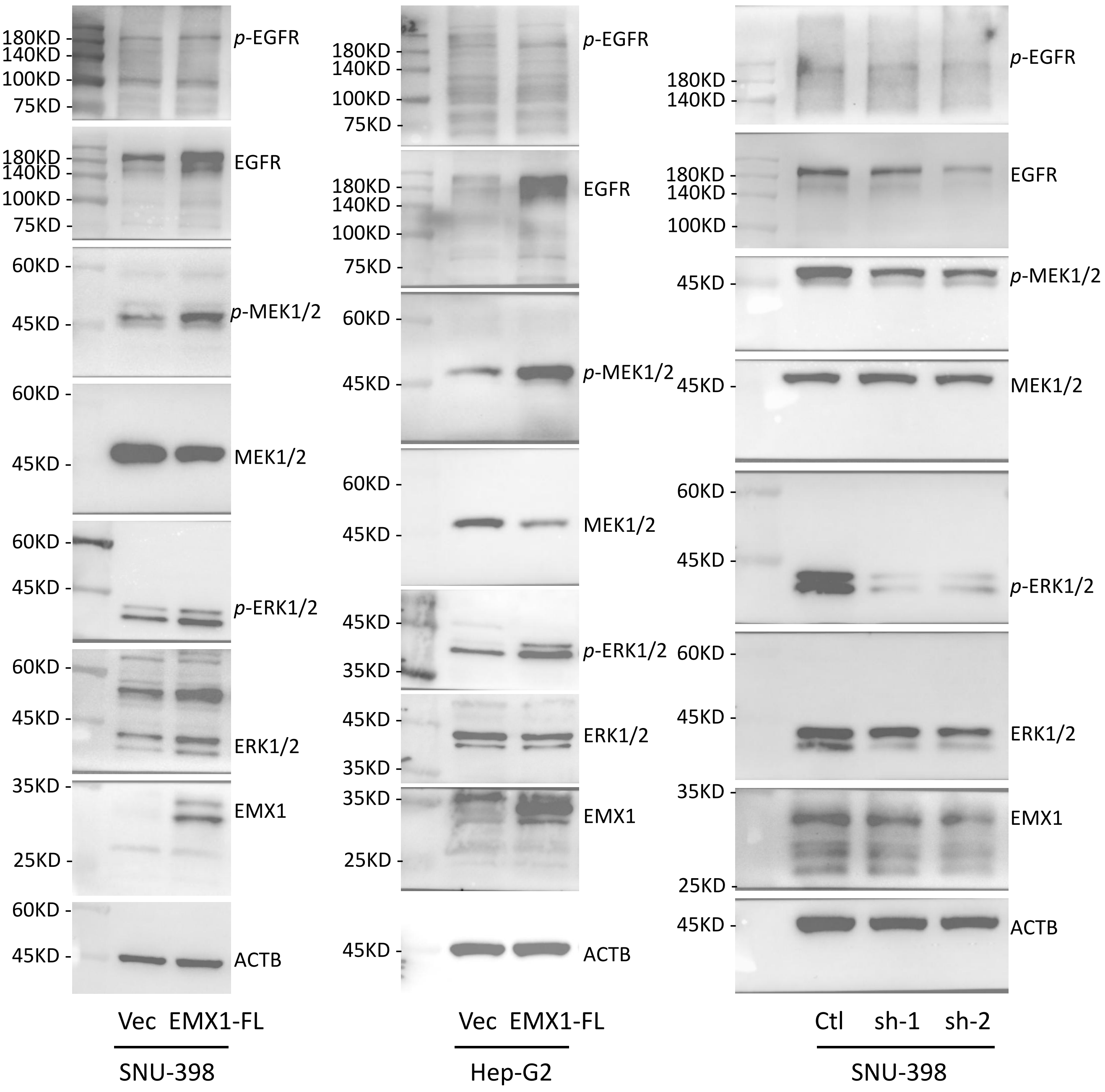
180KD
140KD
100KD
75KD
p-EGFR
EGFR
60KD
45KD
p-MEK1/2
MEK1/2
p-ERK1/2
ERK1/2
35KD
25KD
EMX1
ACTB
Vec EMX1-FL
SNU-398
Vec EMX1-FL
Hep-G2
Ctl sh-1 sh-2
SNU-398

Figure 6B

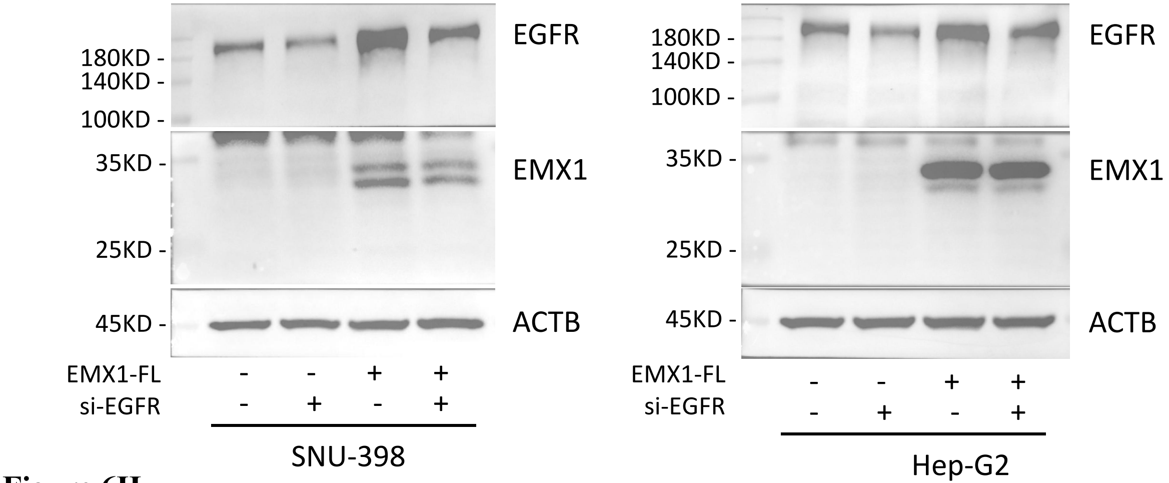

Figure 6H

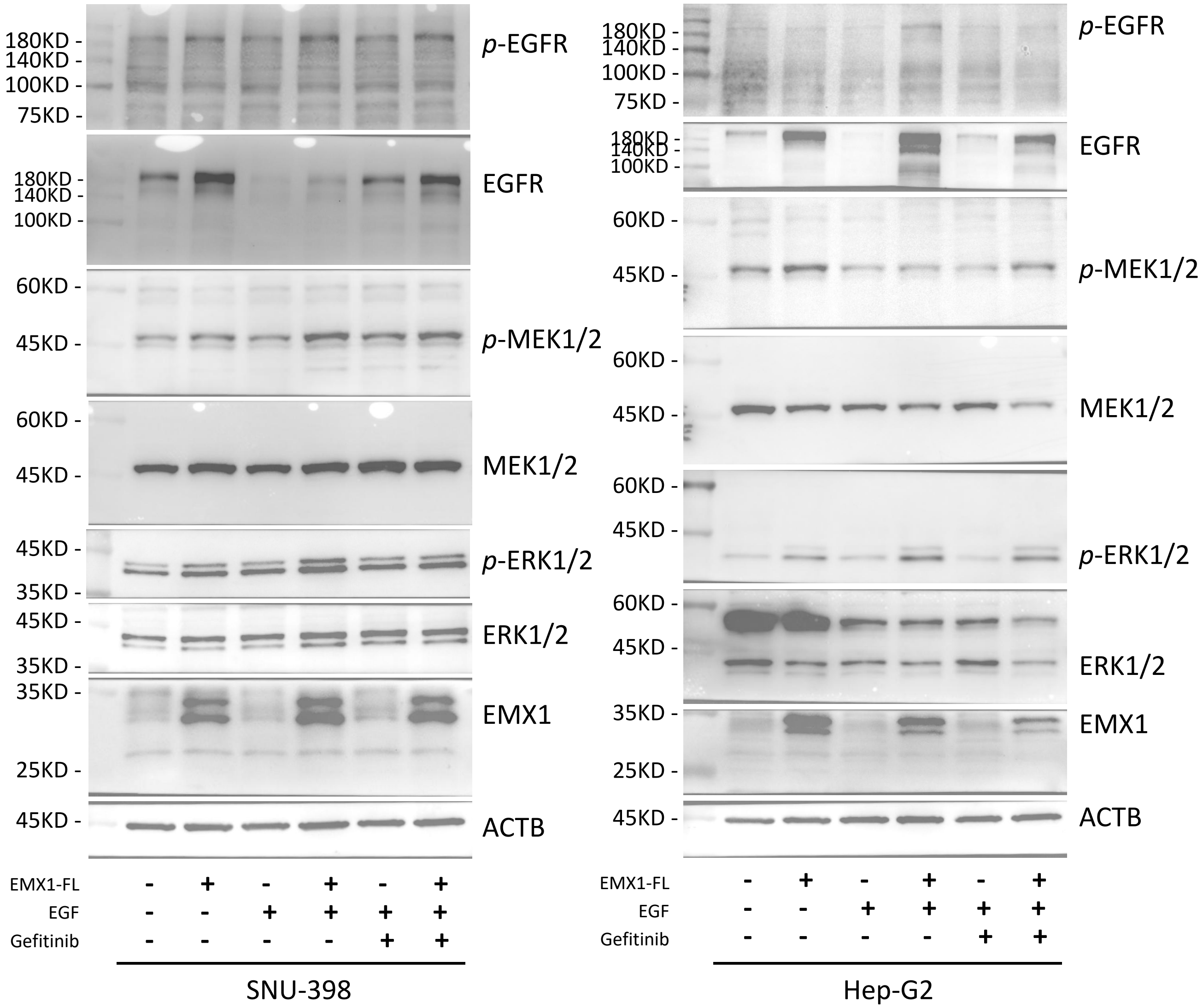

Supplementary Figure 4A

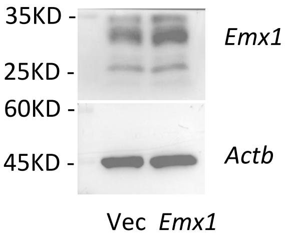

Supplementary Figure 5D

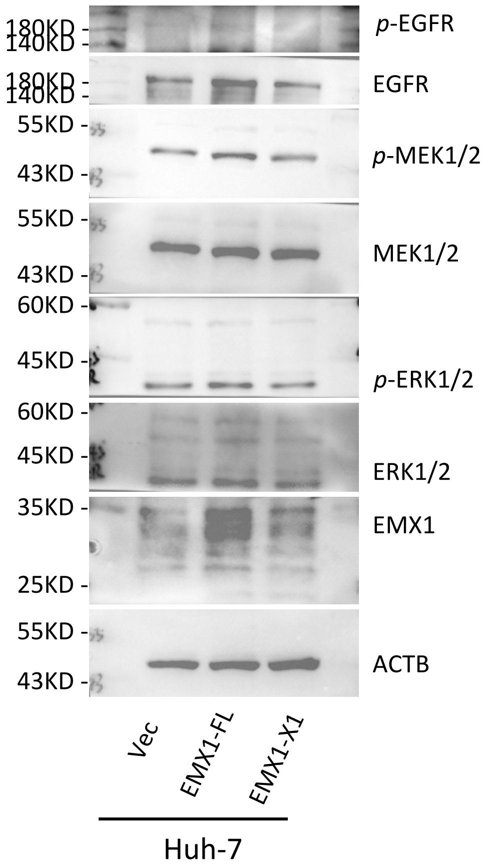

Supplement: Supplementary file 2 — Supplementary File of Western Blot [file 41419_2023_6293_MOESM2_ESM.pdf]
